# Rewiring immunity in rheumatoid arthritis: metabolic and epigenetic therapeutic strategies

**DOI:** 10.3389/fphar.2026.1894277

**Published:** 2026-08-04

**Authors:** Zhiqiang Luo, Shunqian Yao, Zhuquan Zhu, Zehua Chen, Yifan Lu, Wenjie Su

**Affiliations:** 1 Department of Orthopedics, Xiangtan Hospital of Traditional Chinese Medicine, Xiangtan, Hunan, China; 2 Department of Orthopedics, Shaoyang Hospital of Traditional Chinese Medicine, Shaoyang, Hunan, China; 3 Department of Orthopedics, The Orthopedics Hospital of Traditional Chinese Medicine Zhuzhou City, Zhuzhou, Hunan, China; 4 Graduate School, Hunan University of Chinese Medicine, Changsha, Hunan, China; 5 Department of Orthopedics, The Second Affiliated Hospital of Shaoyang University, Shaoyang, Hunan, China

**Keywords:** epigenetic remodeling, immune cells, inflammatory polarization, metabolic reprogramming, metabolism-epigenetics axis, rheumatoid arthritis

## Abstract

Rheumatoid arthritis (RA) is a systemic autoimmune disease marked by chronic inflammation and destruction of joints and surrounding soft tissues. The pathogenesis of RA is heavily driven by two intertwined and mutually regulatory processes: metabolic reprogramming and epigenetic remodeling. Recent advances reveal a crucial crosstalk wherein metabolic intermediates derived from immune cells act as substrates or cofactors for chromatin-modifying enzymes. This interplay profoundly shapes epigenetic landscapes, thereby exacerbating inflammatory responses and disease progression. Here, we review the mechanisms underlying immune cell metabolic reprogramming in RA and critically examine how metabolite-mediated epigenetic remodeling orchestrates disease pathology. Furthermore, we summarize state-of-the-art epigenetic-targeted therapeutic strategies, offering new insights and a solid theoretical basis for treating RA through the metabolic-epigenetic axis.

## 1 Introduction

RA is a chronic systemic autoimmune disease, and persistent synovial inflammation is its main pathological feature (Di Matteo et al., 2023). It is precisely this pathological activation of the synovial membrane that drives the progressive destruction of the joint cartilage and bone tissue (Zheng et al., 2024). This inflammation is typically caused by the infiltration of immune cells such as neutrophils, macrophages, and T cells (TC) (Mondal et al., 2024). Activation of multiple immune cells induces the release of pro-inflammatory mediators, which then initiate and sustain a self-amplifying inflammatory cascade, significantly driving the pathological process of the disease (Wen J. et al., 2025). Additionally, synovial cells and cytokines are involved in joint inflammation (Xu et al., 2022). In the RA synovium, this pathological process is woven by a complex signaling network, encompassing not only multi-dimensional cytokine interactions but also close intercellular crosstalk (Zhang et al., 2025a). RA is clinically characterized by joint swelling, tenderness, and erythema caused by synovial inflammation. If left untreated, the continuous progression of synovial lesions will trigger irreversible structural damage and functional loss (Cutolo et al., 2022). This disease not only manifests as chronic damage to small and medium joints but also indicates systemic inflammatory storms that predict severe extrajoint involvement, including dysfunction of the cardiovascular system and kidneys (Zhang et al., 2024). This multi-system involvement significantly impairs the patient’s mobility and quality of life and is highly associated with metabolic comorbidities such as atherosclerosis, diabetes, obesity, and cardiovascular diseases (Hanlon et al., 2024). Epidemiological data show that the global prevalence of RA is 0.5%–1%, with a particularly high incidence in females. It is noteworthy that RA is not limited to joint pathology; its systemic complications (such as cardiovascular diseases, pulmonary lesions, and anemia) often make the comprehensive management of the disease more challenging (Xu et al., 2025). Epidemiological data indicate that these systemic lesions are significantly correlated with the excess mortality rate of patients (Figus et al., 2021).

Given its steadily rising prevalence and the significant impact it has on patients’ quality of life, RA has become a serious public health challenge (Aghakhani et al., 2022). Currently, treatment strategies for RA focus primarily on pain relief, suppression of synovial hyperplasia, inhibition of vascularisation, modulation of immune homeostasis and cartilage protection, with the ultimate aim of maximising the elimination of inflammation and controlling disease progression (Zhu et al., 2023). At present, the main clinical first-line drugs for RA include disease-modifying antirheumatic drugs (DMARDs), non-steroidal anti-inflammatory drugs (NSAIDs), and glucocorticoids (GCs). Among them, NSAIDs and GCs are mainly used to quickly relieve pain and control acute inflammation, and they are symptomatic treatment drugs. The core drugs that truly have disease-modifying effects and can delay or prevent bone and joint structural damage are DMARDs (Huang et al., 2025). Although conventional therapies can effectively control inflammation, their clinical application is constrained by many factors, including the need for frequent dosing to maintain therapeutic concentrations (which affects patient compliance), as well as the potential cumulative adverse reactions resulting from long-term medication (Zheng et al., 2024). Furthermore, due to insufficient target specificity and limited routes of administration, these drugs are often associated with severe systemic adverse reactions, thereby significantly restricting their clinical application and efficacy (Zhou et al., 2024). In recent years, the emergence of biologics (such as IL-6 inhibitors), small-molecule targeted drugs (such as JAK inhibitors), cell therapies (mesenchymal stem cells, CAR-T cells) and gene-editing technologies (CRISPR-Cas9) has brought about a profound transformation in the treatment landscape of RA. However, whilst these innovative therapies have effectively alleviated symptoms and suppressed inflammatory pathways, frequent disease relapses and pathological rebound reveal their limitations: existing treatment strategies have yet to effectively eliminate underlying immune memory (Tian et al., 2025).

Although the range of therapeutic targets and treatment modalities for RA is becoming increasingly diverse, a significant proportion of patients still fail to achieve clinical remission or low disease activity criteria; this group is clinically defined as refractory RA (D2T-RA) (Nagy et al., 2021). Currently, the clinical management of RA remains a significant challenge: approximately 40% of patients respond poorly to monotherapy, whilst a further 5%–20% exhibit broad drug resistance, leaving them with no available treatment options (Jia et al., 2023). Given the limitations of current therapies in terms of long-term efficacy, there is an urgent clinical need to develop more targeted innovative strategies (Liu W. et al., 2025). Therefore, an in-depth analysis of the molecular mechanisms underlying RA pathogenesis is of paramount importance for refining the existing diagnostic and therapeutic framework and advancing the development of novel targeted drugs (Zhang et al., 2025a).

Currently, RA is regarded as a complex, multifactorial disease driven by immune dysregulation. Although its exact aetiology remains to be fully elucidated, it is generally believed to result from the combined action of internal factors (genetic variations and epigenetic modifications, such as DNA methylation and histone modifications) and environmental factors (such as smoking and pollution) (Aghakhani et al., 2022). Among these, epigenetic modifications (such as DNA methylation and histone modifications) are regarded as a key link connecting the exposome to the onset of autoimmune diseases (Danieli et al., 2024). Furthermore, epigenetics plays a significant role in the prevention, diagnosis and treatment of the disease. Consequently, a thorough understanding of the dynamic evolution of epigenetics and its interactions with other factors is crucial for a comprehensive elucidation of the pathophysiological mechanisms underlying RA (Rojano Rada et al., 2023).

**FIGURE 1:**
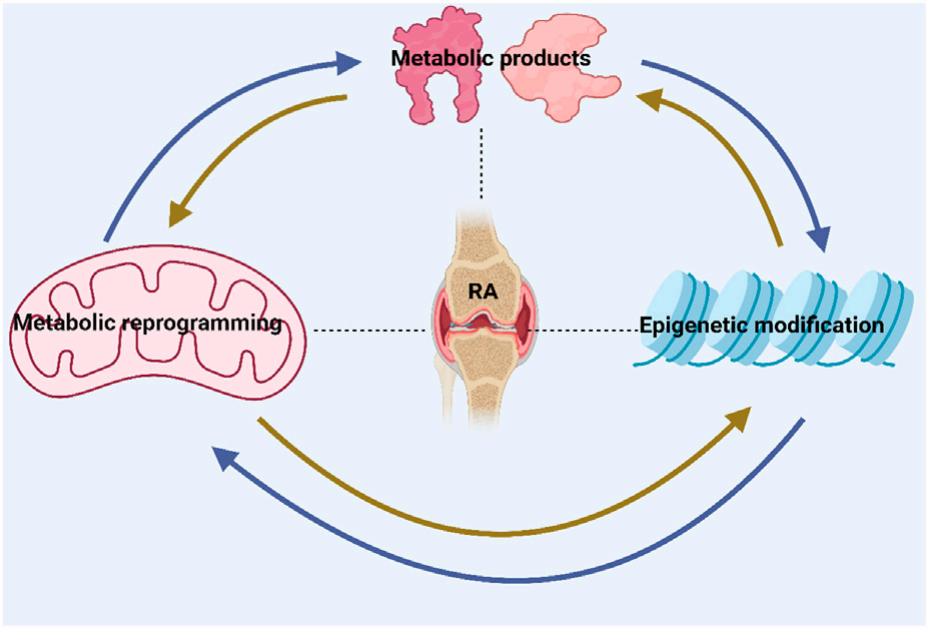
The figure illustrates the bidirectional dialogue between the cellular metabolome and epigenome. During the pathological progression of RA, metabolic reprogramming alters the abundance of metabolic intermediates, which act as substrates or cofactors to directly regulate epigenetic modifications; conversely, epigenetic remodelling can also regulate the transcription of key metabolic enzymes, thereby exerting a feedback effect on cellular metabolic status. A deeper understanding of this three-dimensional, interwoven ‘crosstalk’ network will help elucidate the pathogenesis of RA and provide new strategies for the clinical development of novel combined therapies targeting both metabolic and epigenetic pathways.

Furthermore, metabolic reprogramming, as a core mechanism by which cells adapt to environmental stress, is another key driver of the pathological progression of RA, playing a crucial role in regulating immune cell dysfunction and inflammatory polarisation (Wen J. et al., 2025). Similar to the ‘Warburg effect’ observed in tumours, immune cells within the synovial microenvironment of RA exhibit a unique immunometabolic profile, characterised by the tight coupling of energy metabolism networks with immune functions (such as proliferation, differentiation and cytokine secretion) (Wu et al., 2023; Chen et al., 2025). The emerging field of immunometabolism has revealed metabolic reprogramming occurring in immune cells during activation and differentiation. Through this mechanism, cells adapt their metabolic networks to suit their environment and meet functional demands. Different immune cell subsets possess unique metabolic characteristics, whilst dynamic fluctuations in metabolites serve as key regulatory nodes governing the intensity of the immune response (Liu W. et al., 2025). Immunometabolism is regarded as a key mechanism regulating the initiation and resolution of inflammatory responses, profoundly influencing the pathological progression and outcomes of infectious and autoimmune diseases. Understanding the role of immunometabolism in the regulation of inflammation is of paramount importance (Pomeyie et al., 2025). Research into immunometabolism has revealed the core driving mechanisms of metabolic imbalance in disease progression by analysing the interactive networks between metabolic profiles and immune responses (Zhang et al., 2025b). As a hub for environmental sensing, the metabolic state is deeply involved in the remodelling of the epigenetic landscape: intermediate metabolites mediate precise regulation of chromatin dynamics and gene expression by inducing post-translational modifications (PTMs) of chromatin (Sun et al., 2022). In the pathological microenvironment of RA, this metabolic-epigenetic coupling mechanism induces significant metabolic reprogramming in macrophages, disrupting the M1/M2 polarisation homeostasis and thereby triggering and sustaining a persistent inflammatory cascade (Zheng et al., 2024). In summary, a bidirectional regulatory mechanism exists between cellular metabolic reprogramming and the epigenome, and both are intertwined and mutually influence metabolic intermediates in RA (Figure 1).

This review aims to systematically elucidate the metabolic reprogramming and functional remodelling of key pathogenic cells—including fibroblast-like synoviocytes (FLS), macrophages and T cells—in RA, and to explore in depth metabolite-driven epigenetic modifications and their mediated pathological effects.

Although previous literature has explored metabolic dysregulation and epigenetic abnormalities in RA in depth, most studies still treat them as parallel pathological phenomena, or confine metabolites to the role of ‘static substrates’ whilst viewing epigenetics as a ‘stable regulatory barrier’. To address this limitation, this review proposes a systemic perspective that integrates the two into a ‘dynamic self-driving axis’, aiming to answer a core scientific question: is metabolic reprogramming a passive by-product of the inflammatory response, or is it a key driver that ‘consolidates’ the inflammatory phenotype by reshaping chromatin dynamics? The academic breakthrough of this study lies in defining metabolites, for the first time, as ‘translators of epigenetic remodelling’, systematically demonstrating how local microenvironmental stresses in the synovium (such as hypoxia and metabolic deprivation) are converted into permanent chromatin modification marks via a metabolic-epigenetic feedback loop, thereby elucidating the mechanism underlying the transformation of RA synovial fibroblasts (RASFs) from a ‘reactive’ to a ‘self-driving’ malignant phenotype. This metabolically induced ‘metabolic-epigenetic memory’ constitutes the underlying logic behind the chronic nature of the disease. Building on this, this paper not only aims to elucidate how this coupling mechanism disrupts immune homeostasis, but also further evaluates the translational potential of intervening in this feedback loop using ‘epimetabolic drugs’, thereby providing conclusive mechanistic evidence and new strategies for precision therapy to combat refractory RA (D2T-RA).

## 2 The metabolic rewiring landscape in RA

RA, an immune-mediated disease with complex pathophysiology, is significantly influenced by disruptions in metabolic networks. The scientific community is actively investigating how glycolysis, the tricarboxylic acid (TCA) cycle, the pentose phosphate pathway (PPP), arachidonic acid (AA) metabolism and amino acid metabolism interact in RA, and has made significant progress in elucidating the underlying molecular mechanisms. As a chronic autoimmune disease, research indicates that cellular metabolic reprogramming plays a central regulatory role in the complex pathogenesis of RA (Jia et al., 2023). The awarding of the 2019 Nobel Prize in Physiology or Medicine profoundly revealed the cornerstone role of cellular oxygen sensing and adaptation mechanisms in biology. This mechanism is closely intertwined with energy metabolism, together forming the axis that regulates physiological balance and pathological progression in the body. The phenomenon of aerobic glycolysis, exemplified by the Warburg effect, is not only a hallmark of metabolic reorganisation in tumour cells under oxygen-rich conditions but has also been shown to be extensively involved in various pathological processes, including autoimmune diseases, sepsis, diabetes and ageing, reflecting commonalities in metabolic adaptation across disease dimensions (Nguyen et al., 2016). A hallmark feature of aerobic glycolysis (also known as the Warburg effect) is a significant increase in cellular glucose uptake, with metabolic flux prioritising lactate production, even in environments with ample oxygen supply (Lu Y. et al., 2024). Glycolysis is the central pathway for ATP production in organisms under anaerobic conditions, and also serves as a key link connecting aerobic and anaerobic pathways in energy metabolism. This process first breaks down glucose into pyruvate, the subsequent metabolic fate of which is oxygen-dependent: in the presence of ample oxygen, it enters the mitochondria for complete oxidation; whereas in hypoxic conditions, it is converted into lactate via reductive reactions (Ao-Di et al., 2024). In-depth studies reveal significant metabolic reprogramming in the inflamed synovial tissue of RA: abnormally upregulated glycolytic flux disrupts the balance of the microenvironment, manifesting as substrate depletion due to rapid glucose consumption and pathological accumulation of the by-product lactate (Qiu et al., 2021).

Furthermore, clinical metabolomic analyses indicate that synovial fluid from RA patients exhibits distinct features of glucose deprivation and lactate accumulation (Yang et al., 2015), resulting in a characteristic acidic microenvironment. Lactate plays a key role in catalysing the development of RA (Liu W. et al., 2025). Transmembrane transport of lactate is primarily mediated by monocarboxylate transporters (MCTs), which facilitate the shuttling of lactate between the intracellular and extracellular compartments. Notably, this transport process is synergistically regulated by pH, the lactate concentration gradient, and the cellular redox state (Sun et al., 2017; Ye et al., 2022). As lactate accumulates and is released into the extracellular environment, it is taken up by CD4^+^ T cells, macrophages, dendritic cells and osteoclasts via lactate transporters in a concentration-dependent manner. This uptake process induces the differentiation and activation of these cells, ultimately exacerbating the pathological progression of RA by reshaping their biological functions (Haas et al., 2015; Shen et al., 2017).

The glycolytic pathway is strictly regulated by three key rate-limiting enzymes: hexokinase (HK), phosphofructokinase-1 (PFK-1) and pyruvate kinase (PK). These enzymes constitute the primary control points in the metabolic cascade (Hu et al., 2025). Hypoxia-inducible factor 1α (HIF-1α) is the primary regulator of cellular adaptation to hypoxia (Li et al., 2025). Under the dual influence of the hypoxic environment and reactive oxygen species (ROS) accumulation in synovial tissue, the HIF-1α protein remains stable and undergoes nuclear translocation, thereby initiating the transcription of downstream genes (Cummins et al., 2016). As a central transcriptional regulator of glycolysis, HIF-1α remodels cellular metabolic pathways through synergistic multi-target actions. On the one hand, it directly upregulates the expression of glucose transporters (Glut1) and key rate-limiting enzymes (Hk2, Pkm2) to ensure energy supply under hypoxic stress; on the other hand, HIF-1α promotes lactate production by inducing lactate dehydrogenase A (LDHA) and synergistically inhibits mitochondrial oxidative phosphorylation (OXPHOS), thereby facilitating a metabolic phenotypic switch from oxidative phosphorylation to glycolysis (Yang et al., 2023; Meng et al., 2025). Genome-wide analyses indicate that HIF-1α acts as a key epigenetic regulator, capable of precisely modulating hundreds of RA-associated disease-causing genes. Its influence spans multiple pathological dimensions, with deep intervention observed across a range of processes, from angiogenesis and metabolic reprogramming in synovial tissue to inflammatory cascades (such as cytokine production and citrullination), as well as the invasion and migration of fibroblasts and the regulation of intestinal barrier function (Kalliolias and Papavassiliou, 2024).

Synovial inflammation in RA is sustained by interactions between T cells, macrophages and FLSs(Zhu et al., 2023). These immune cells are deeply coupled with matrix cells through complex molecular crosstalk, working in concert to shape a persistent pro-inflammatory microenvironment, which in turn mediates the progressive destruction of joint tissue (Sekkout et al., 2024). The pathogenesis of RA is highly dependent on functional abnormalities in synovial resident cells (such as resident macrophages and FLSs). Studies have shown that the metabolic profiles of these cells undergo fundamental changes; this metabolic reprogramming not only reshapes cellular phenotypes but is also a key driver of the disruption of synovial tissue homeostasis (Zhang et al., 2025a).

Synovial cells—particularly activated FLS—have been identified as the key effector cells mediating structural damage and destruction in RA (José Alcaraz, 2021). Studies have confirmed that RASFs exhibit significant ‘tumour-like’ biological behaviour, with their lack of contact inhibition and enhanced resistance to apoptosis jointly driving the pathological proliferation of synovial tissue and joint erosion. As hubs of angiogenesis, RA FLS directly induce cartilage degradation through the high-level secretion of pro-inflammatory cytokines and matrix metalloproteinases. These findings establish RASFs as central drivers of RA pathogenesis, completely overturning the outdated notion that they were previously regarded as “passive bystanders” (Aghakhani et al., 2022). In patients with RA, FLSs undergo significant metabolic reprogramming, shifting from oxidative phosphorylation to glycolysis. This metabolic shift leads to abnormal proliferation, resistance to apoptosis and enhanced invasiveness in FLSs (Liu W. et al., 2025). Experimental data indicate that MET stimulation significantly enhances the pro-inflammatory and invasive phenotypes of RA-FLSs: compared with untreated controls, the expression levels of pro-inflammatory cytokines (TNF and IL-1β) and matrix-degrading enzymes (MMP-9 and MMP-13) show a significant upward trend (Weng et al., 2024). In this pathological process, the accumulation of reactive oxygen species (ROS) induced by impaired mitochondrial function became the key trigger for the activation of the HIF-1α/Notch-1 signalling axis. Activation of this signalling pathway further induces high expression of matrix metalloproteinases (MMP-3/9) and vascular endothelial growth factor (VEGF), thereby directly driving synovial hyperplasia and cartilage erosion. Furthermore, the pro-inflammatory microenvironment established by synovial cells maintains cellular phenotypic transformation through a positive feedback loop, creating a self-perpetuating inflammatory vicious cycle that significantly exacerbates the clinical symptoms of RA (Fearon et al., 2022; Zhang et al., 2025a). Existing research confirms that the metabolic disturbances of glucose, lipids and amino acids occurring within FLS in RA are the core factors driving pathological synovial hyperplasia and inflammatory cascades. In particular, the abnormal activation of the glycolytic pathway not only constitutes a key metabolic feature of RA but is also finely regulated by the transcription factor HIF-1α. Consequently, targeting HIF-1α to restore metabolic homeostasis has emerged as a highly promising new strategy in the field of RA treatment in recent years (Zhu et al., 2023).

Research into immunometabolism has revealed that the activation and differentiation of immune cells are heavily dependent on metabolic reprogramming. This metabolic transformation is not merely a passive adaptation to nutritional pressures in the microenvironment, but rather an autonomous adjustment undertaken by cells to precisely meet the bioenergetic and synthetic requirements of specific effector functions. By altering metabolic flux and the abundance of key metabolites, different immune subsets exhibit unique metabolic profiles, thereby determining the intensity and polarisation of the immune response at a fundamental level (Chen et al., 2024; Liu W. et al., 2025). Metabolic reprogramming of immune cells is closely associated with the development and progression of autoimmune diseases (Wang et al., 2024). Macrophages are regarded as central effector cells of the immune system, playing an irreplaceable pivotal role in maintaining tissue homeostasis, initiating immune responses, and regulating the resolution of inflammation. Macrophages exhibit remarkable phenotypic plasticity, capable of undergoing directed polarisation in response to variations in microenvironmental signals: they are primarily classified into the classically activated (M1) type, which possesses pro-inflammatory characteristics, and the alternately activated (M2) type, which combines anti-inflammatory and tissue-repair functions (Peng et al., 2023). This polarised state determines whether they act as ‘destroyers’ or ‘repairers’ within the cytokine network of RA (Yu et al., 2025). Driven by specific microenvironmental signals, macrophages undergo directed polarisation and adopt a pro-inflammatory phenotype. Through the massive secretion of IL-1β, IL-12, IL-6, TNF-α and various chemokines, these cells effectively construct a network of inflammatory mediators. This secretory profile synergistically triggers the recruitment and activation of effector T and B cells, thereby generating an amplifying effect that significantly exacerbates the pro-inflammatory microenvironment within the joint cavity (Zhang et al., 2024).

Macrophages are widely recognised as a central hub in the pathological process of RA. Their pathogenic role is primarily manifested in the disruption of the balance between M1 (pro-inflammatory) and M2 (anti-inflammatory) polarisation within the synovial microenvironment; this shift in polarisation directly drives a sustained inflammatory cascade (Yan et al., 2024). It should be noted that although the binary M1/M2 classification provides a basic conceptual framework for understanding macrophage polarization, it overly simplifies the complex phenotypic spectrum observed in the synovium of human RA. Therefore, recognizing this heterogeneity is crucial, as the pathogenic role of these cells in RA is not merely the result of a simple M1/M2 transformation, but stems from complex, environment-dependent metabolic and epigenetic adaptation mechanisms.

As key effector cells mediating joint damage, their clinical significance is equally profound: the infiltration density of sublining macrophages has become a recognised biomarker for assessing RA disease activity and predicting treatment response (Hanlon et al., 2024). In the pathological progression of RA, pro-inflammatory M1 macrophages play a dominant role; through the high expression of surface markers CD80/CD86 and the release of inflammatory mediators such as TNF-α, IL-1β and CXCL9, they serve as the core driving force behind joint pathology. In contrast, M2 macrophages are characterised by the expression of CD206, CD209 and CD163, as well as the upregulation of IL-10, and perform anti-inflammatory and tissue-repair functions. Consequently, driving the phenotypic transition of synovial macrophages from M1 to M2 (i.e., immune reprogramming) has emerged as a highly promising strategic direction for restoring immune homeostasis and treating RA (Zhou et al., 2024). The inflammatory activation of M1 macrophages is accompanied by a global shift in metabolic patterns. By upregulating key metabolic enzymes such as hexokinase II (HKII) and glucose-6-phosphate dehydrogenase (G6PD), cells significantly enhance glycolytic flux, ensuring energy supply for the execution of effector functions. Concurrently, the TCA cycle undergoes programmed disruption at specific sites, leading to the intracellular accumulation of intermediate metabolites such as succinate and citrate. Fluctuations in these metabolites, together with surging lactate production, constitute the metabolic signature of M1 cells and essentially initiate and sustain the inflammatory cascade (De Jesus et al., 2022; Yan et al., 2024). Studies have shown that, in the pathological context of RA, the ERK/HIF-1α/GLUT1 signalling axis has been identified as the core mechanism driving M1 macrophage polarisation (Umar et al., 2021; Yan et al., 2024). At the molecular level, the pathological accumulation of metabolic intermediates such as succinate (SA) and citrate (CA) effectively stabilises the HIF-1α protein, thereby enhancing the transcriptional activity of M1 effector factors. Subsequently, the enhanced expression of HIF-1α not only upregulates glucose transporter 1 (GLUT1) but also directly induces the transcription of key glycolytic enzymes such as lactate dehydrogenase A (LDHA), hexokinase II (HKII) and pyruvate kinase M2 (PKM2). This metabolic reprogramming, mediated by the signalling axis, forms a positive feedback loop for the pro-inflammatory phenotype, significantly exacerbating the clinical progression of RA (Kim et al., 2022).

Consequently, in the pathophysiology of RA, the imbalance between pro-inflammatory M1 and anti-inflammatory M2 macrophages not only disrupts immune homeostasis but also reflects a deeper level of intracellular metabolic reprogramming: namely, the pathological shift of M1 macrophages towards glycolysis and the preference of M2 macrophages for oxidative phosphorylation. Consequently, intervening in metabolic pathways to reverse this shift in macrophage polarisation has emerged as a highly promising therapeutic approach for restoring immune balance and alleviating joint inflammation (Zheng et al., 2024).

Furthermore, T cells constitute the predominant population of immune cells infiltrating the synovial tissue in RA (Zhu et al., 2023). As the linchpin of the adaptive immune system, T cells play an indispensable role in reshaping the body’s homeostasis and establishing a multidimensional defence system against disease by precisely mediating cellular immune responses (Sun et al., 2023). T cells originate from developmental selection in the thymus and primarily differentiate into two major lineages: CD8^+^ cytotoxic T cells and CD4^+^ helper T cells. Among these, CD4^+^ T cells exhibit exceptional multi-directional differentiation potential; under the induction of specific microenvironmental signals, they can evolve into functional subsets such as Th1, Th2, Th9, Th17, Tfh and Treg. Each subset precisely regulates different types of immune responses through its unique cytokine secretion profile (Golubovskaya and Wu, 2016). Under physiological conditions, the numbers and distribution of peripheral T cells and their functional subsets maintain a precise dynamic equilibrium. Any deviation in the total number of T cells, the absolute count of specific subsets, or their relative proportions signals a disruption of immunological homeostasis. This disruption of immunological homeostasis is widely recognised as a core pathological basis mediating the onset and progression of numerous complex diseases (Adu-Berchie et al., 2023). In addition to traditional Th1 and Th17 cells initiating disease through the release of key inflammatory mediators (such as IL-1 and IL-17), recent evidence further suggests that Th22 cells also play a significant role in driving the pathological progression of RA (Zhu et al., 2023). Furthermore, non-conventional T cells, such as NKT, MAIT and γδT cells, have emerged as key hubs regulating the local immune microenvironment in RA through the release of pro-inflammatory factors (e.g., IFN-γ, TNF-α and IL-17) and extensive intercellular communication (involving synovial cells, dendritic cells, B cells and osteoclast precursor cells), thereby profoundly mediating the inflammatory and destructive processes in the joints (Xu et al., 2025).

T cells drive the pathological progression of RA through two synergistic pathways: firstly, by triggering an immune response through antigen recognition, which induces the activation of effector cells and the release of a vast array of cytokines (including IL-6, IL-17 and TNF-α). Secondly, disruption of the Th17/Treg axis destabilises immune homeostasis, thereby initiating and sustaining a persistent inflammatory cascade that leads to progressive destruction of joint tissue (Anandan and Narayanan, 2025). Although Th17 cells and Treg cells share a common lineage originating from naive CD4^+^ T cells, they exhibit significant antagonistic characteristics in their functional evolution. As the core of the pro-inflammatory axis, Th17 cells primarily mediate the cascade amplification of inflammatory damage. In contrast, Treg cells serve as key guardians of immune tolerance; by secreting anti-inflammatory factors and directly interfering with Th17 effector functions, they establish a balancing mechanism that maintains the body’s immune homeostasis (Paradowska-Gorycka et al., 2020). Research has confirmed that Treg cells, by enhancing the expression of the key transcription factor Foxp3, can effectively suppress the transcriptional levels of IL-23 and IL-17, thereby blocking the differentiation process of Th17 cells at the molecular source. This regulatory mechanism exhibits significant reciprocity: the suppression of the Th17 pathway relieves the pressure it exerts on the suppressive immune response, thereby creating a favourable microenvironment for Treg cell development. This phenomenon profoundly reveals the antagonistic and dynamic relationship between the two in terms of their differentiation fates (Fujio et al., 2010; González-Osuna et al., 2022).

An imbalance in the Th17/Treg cell population is regarded as a key determinant driving the progression of RA, playing a central role in mediating the amplification of inflammation within the local microenvironment. Clinical evidence indicates that the frequency of Th17 cells and their effector cytokines (such as IL-17, IL-23, IL-6 and TNF-α) in the peripheral blood of RA patients is abnormally elevated; concurrently, Treg cells, which possess immunosuppressive functions, and their key factor—transforming growth factor-β (TGF-β)—are significantly downregulated (Park et al., 2022). This pathological immune polarisation has also been validated in the patient bone marrow microenvironment, manifesting as the synergistic expansion of Th1 and Th17 populations and a significant reduction in Treg frequency (Wang et al., 2016). In the pro-inflammatory microenvironment of the synovium in RA, regulatory T cells (Tregs) are often reduced in number or functionally impaired. The core mechanism lies in the fact that high levels of pro-inflammatory cytokines (such as IL-1β and IL-6) significantly impair the stability and expression of the key transcription factor Foxp3, thereby depriving Tregs of their immunosuppressive efficacy and making it difficult for them to curb the inflammatory cascade within the synovium (Li and Zheng, 2015; Jiang et al., 2021). The differential expression of HLA-DRB1 and PTPN-2 in patients further consolidates their status as core pathogenic factors in RA. Within the pathological microenvironment, the metabolic homeostasis of T cells (TCs) is subject to multidimensional regulation by cytokines, mitochondrial proteins and complex metabolic products. Crucially, different T cell subtypes drive the programmed transition from naive T cells (T0) to an effector-activated state through metabolic reprogramming—that is, the flexible switching between glycolysis, oxidative phosphorylation or fatty acid oxidation (FAO) modes according to functional requirements (Mondal et al., 2024). Regulatory T cells (Tregs) exhibit unique bioenergetic characteristics in their activated state, maintaining energy homeostasis primarily through TCA cycle and FAO. In stark contrast, pro-inflammatory effector subsets such as Th1 and Th17 undergo profound metabolic remodelling, shifting towards an aerobic glycolysis-dominated metabolic mode accompanied by significant lactate accumulation. This differential selection of metabolic platforms clearly reveals a deep coupling between cellular metabolic lineages and T-cell effector functions: specific metabolic fluxes not only ensure energy supply but also serve as a core mechanism for the fine-tuning of cytokine synthesis profiles and secretory polarisation (Lopez Krol et al., 2022). Consequently, the precise regulation of T-cell activation phenotypes through the targeting of their bioenergetic metabolism has emerged as a key therapeutic strategy for restoring immune homeostasis in RA.

**FIGURE 2:**
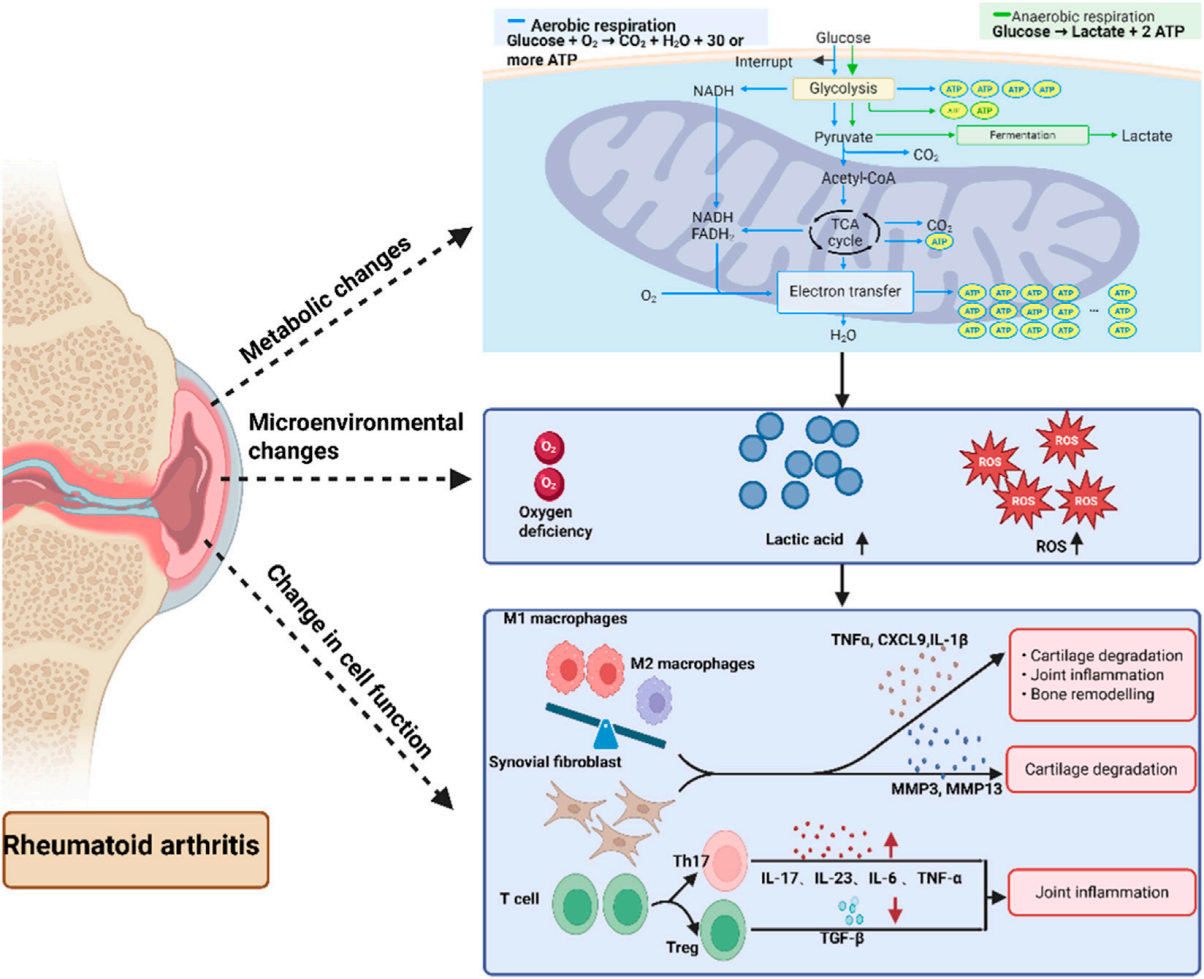
The figure shows that RA lesions exhibit microenvironmental characteristics such as hypoxia, lactate accumulation and ROS overload, accompanied by a shift in cellular metabolism from oxidative phosphorylation to glycolysis. This pathological microenvironment further drives phenotypic shifts in effector cells: including an imbalance between Th17/Treg and M1/M2 macrophages (with an increase in pro-inflammatory cells and a decrease in anti-inflammatory cells), as well as abnormal proliferation of fibroblasts, which leads to the degeneration and erosion of cartilage.

In summary, the metabolic remodelling of T lymphocytes, macrophages and FLSs in the RA synovial microenvironment constitutes the underlying mechanism driving cellular phenotypic polarisation and functional activation, and ultimately governs the pathological progression of the disease (Figure 2). In this regard, elucidating and intervening in the metabolic programmes of these core cell populations, with a view to reversing the pathological state by correcting energy metabolism imbalances, will provide a solid biochemical foundation and new perspectives for clinical intervention in the development of precision treatment strategies for RA.

However, although there is currently a consensus within the academic community that ‘HIF-1α-mediated glycolytic reprogramming underlies synovial invasion’, the field still faces significant translational challenges: on the one hand, multiple clinical metabolomic analyses have demonstrated significant metabolic heterogeneity among RA patients, suggesting that a ‘one-size-fits-all’ glycolytic inhibition strategy may face limitations in complex clinical populations; on the other hand, a simple increase in glycolytic flux often fails to fully account for the complex invasive phenotype of FLSs. This mechanistic contradiction suggests that metabolic reprogramming in RA exhibits a high degree of cell-type specificity and may involve more refined regulatory mechanisms. Therefore, future research should move beyond the limitations of ‘overall concentration’ and focus on the epigenetic spatial distribution of key metabolites within specific gene promoter regions, thereby providing a more targeted mechanistic basis for achieving precise metabolic interventions in RA.

## 3 The epigenetic architecture of RA

Epigenetics refers to the regulation of gene expression through heritable and reversible mechanisms without altering the primary sequence of DNA, thereby producing phenotypic changes independent of DNA sequence variations (Rojano Rada et al., 2023). Since the concept was first introduced in 1942, research in epigenetics has made leaps and bounds thanks to advances in high-throughput detection technologies. As a central hub linking environmental signals to gene expression, epigenetic modifications transcend the fixed constraints of genomic sequences, demonstrating unique dynamic plasticity and reversibility. Their pathophysiological functions are primarily realised through three synergistic mechanisms: DNA methylation, histone modifications, and non-coding RNA regulation (Su et al., 2025). Epigenetic modifications act as a ‘regulatory hub’ for gene expression and exhibit extreme sensitivity to environmental stimuli—factors such as environmental pollution, dietary changes, psychological stress and pathogen invasion can all induce alterations in these modifications. In the progression of autoimmune diseases, pathological epigenetic reprogramming often leads to the collapse of immune tolerance and dysregulation of the immune response; the resulting secretion of autoantibodies is a key mechanism underlying tissue damage (Danieli et al., 2024). Epigenetic regulatory networks are constructed through the synergistic action of three classes of core functional proteins: “Writers” act as donors of modifications, responsible for attaching chemical tags to DNA or histones; “Readers” act as effector molecules, decoding these modification signals via specific recognition domains; whilst “Erasers” remove tags through hydrolysis or demodification, thereby enabling the dynamic resetting of genetic signals (E K et al., 2025). Extensive research has confirmed that disruptions in the epigenetic regulatory system induce pathological signalling aberrations, manifesting specifically as abnormal establishment (Writing), misrecognition (Reading) or impaired clearance (Erasing) of epigenetic marks, thereby disrupting the homeostasis of gene expression (Glant et al., 2014).

**FIGURE 3:**
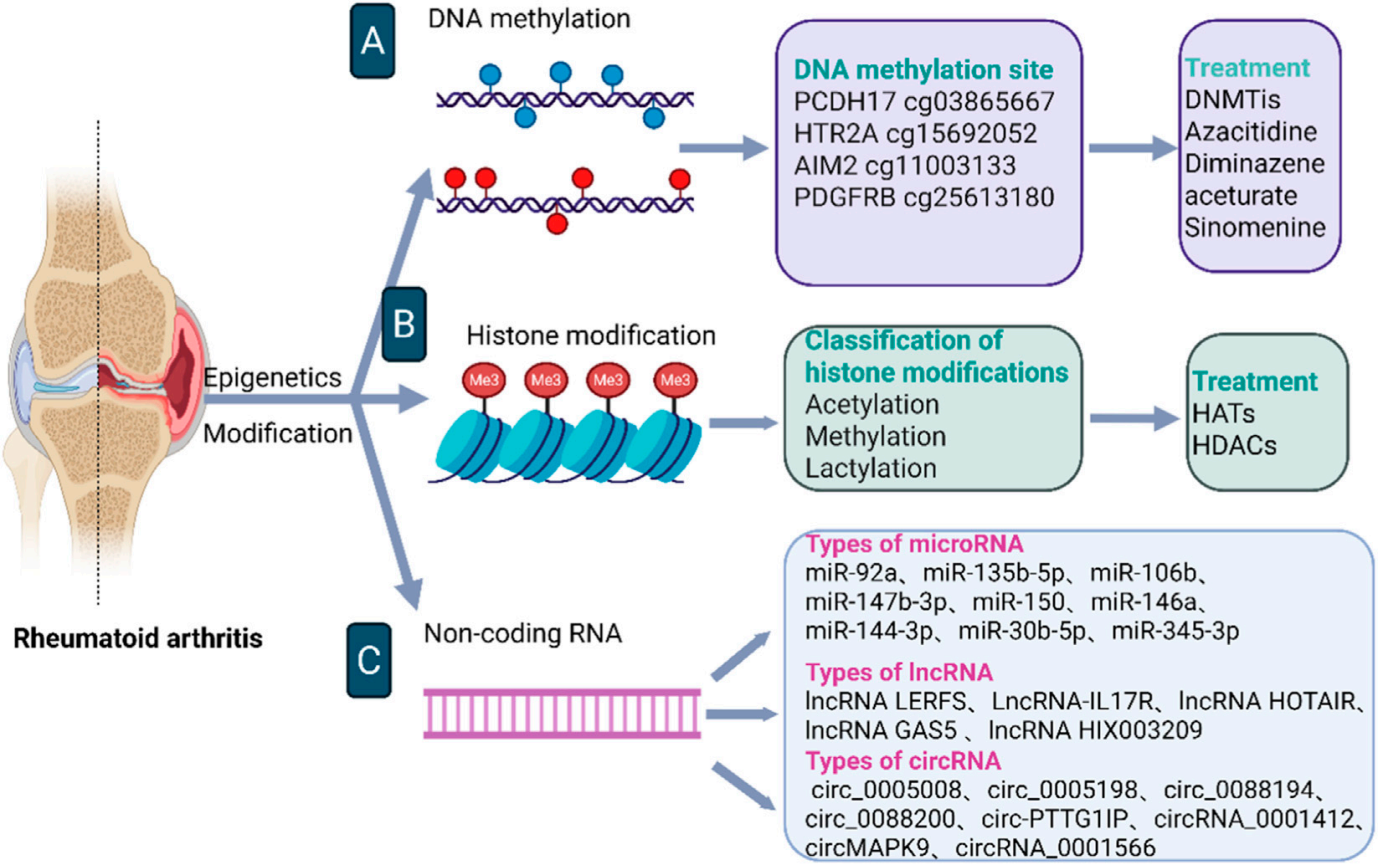
Epigenetic subtypes, pathogenic mechanisms and therapeutic strategies in rheumatoid arthritis. **(A)** DNA methylation: Illustrates abnormalities in RA-associated specific DNA methylation sites and strategies for targeted intervention. **(B)** Histone modifications: Elucidates the systematic classification of histone modifications and therapeutic strategies targeting specific modifying enzymes. **(C)** Non-coding RNA types: Provides a detailed overview of the molecular networks of microRNAs, lncRNAs and circRNAs that regulate the progression of RA.

RA is the most common form of autoimmune arthritis, characterised primarily by synovial inflammation (Baghel et al., 2025). Its aetiology is usually attributed to specific genetic variants (particularly HLA alleles) (Srivastava and Rasool, 2024). However, a growing body of evidence highlights the highly complex nature of the pathogenesis of RA, and it is difficult to fully explain its heterogeneous phenotype through a single genetic variant alone (Chen et al., 2022). Cutting-edge evidence indicates that epigenetics plays a key role in the formation and organisation of the immune response in RA (Vijaykrishnaraj et al., 2023). These epigenetic alterations play an indispensable role in driving the complex pathological evolution of RA (Srivastava and Rasool, 2024). Existing research increasingly confirms that the pathological landscape of RA is jointly shaped by genetic background and the epigenetic landscape. Epigenetic modifications not only reshape the gene expression profile of cells but also induce cascading disruptions in key signalling pathways and dysregulation of the immune response; this non-sequence-dependent regulatory mechanism has become a pivotal hub driving the progression of RA (Zhu et al., 2023). Similar to most autoimmune diseases, RA is a complex pathological process driven by a combination of genetic background, environmental stressors and endogenous signalling. The core mechanism of these triggers lies in the profound remodelling of the epigenetic landscape of pathogenic cells (Rojano Rada et al., 2023). Consequently, the systematic elucidation of the pathological mechanisms of epigenetic regulation in RA has become a priority in current immunological research (Figure 3).

### 3.1 DNA methylation

DNA methylation is a biochemical modification process mediated by DNA methyltransferases (DNMTs). Its core mechanism involves the precise transfer of a methyl group to the 5′ end of the cytosine base within CpG sites (i.e., cytosine-phosphate-guanine sequences) (Rojano Rada et al., 2023). DNA methylation is not only the most common epigenetic modification in the mammalian genome, but also the most well-established and deeply studied regulatory mechanism in the current field of epigenetics (Su et al., 2025). DNA methylation is an indispensable epigenetic ‘switch’ in living organisms; it not only governs gene expression and growth and development, but also plays a decisive role in the ageing process and the onset and progression of various malignant diseases (Fu et al., 2025).

RA is driven by a combination of genetic, environmental and epigenetic factors. In this process, DNA methylation acts as a key regulator of the body’s inflammatory and immune responses by modifying immune genes (Mucientes et al., 2025). Previous studies have confirmed that abnormal DNA methylation patterns emerge in the early stages of rheumatic diseases, suggesting that they may be involved in the initiation of the disease (Krishna Priya et al., 2025). Following decades of research, studies on DNA methylation in both clinical and animal models of RA consistently indicate that genome-wide hypomethylation is a highly representative biochemical marker of RA (Kiełbowski et al., 2025). Whole-genome DNA methylation sequencing of peripheral blood mononuclear cells (PBMCs) from patients with RA revealed that epigenetic regulation drives the progression of RA by mediating the formation of an interferon-induced gene interaction network. Among these, the PARP9 gene was identified as a central node in this pathogenic mechanism (Chen et al., 2022). Low methylation of the PARP9 promoter not only demonstrates excellent diagnostic discriminatory power for SLE and RA, but its high correlation with disease activity indices also suggests that this epigenetic feature has great potential as an alternative marker for reflecting disease progression and clinical monitoring (Sohanforooshan Moghaddam et al., 2025).

As a key epigenetic driver of autoimmune diseases, dysregulated DNA methylation induces the breakdown of immune tolerance and subsequent immune attacks by disrupting gene expression patterns. This critical pathogenic mechanism confers significant biomarker value for precise diagnosis and monitoring of disease progression (Ciechomska et al., 2019; Nair and Wilson, 2023). Although the traditional diagnostic framework for RA centres on clinical manifestations and rheumatoid factor (RF) and anti-cyclic citrullinated peptide (ACPA) antibodies, it is limited by insufficient sensitivity and specificity, leading to clinical challenges such as missed diagnoses in seronegative cases or false-positive misdiagnoses. Consequently, the identification of highly reliable novel biomarkers has become an urgent requirement for achieving precision medicine in RA (Mohamadizad Moghadam et al., 2025). DNA methylation, as a potential diagnostic marker for RA, shows excellent prospects for clinical application. Given that early intervention is a decisive factor in optimising the long-term prognosis of RA patients, the use of epigenetic markers to achieve early diagnosis and treatment is of significant strategic importance (Huang et al., 2024). Multiple epigenome-wide association studies (EWAS) have identified differentially methylated sites (DMPs) and regions (DMRs) closely associated with RA. These epigenetic variations are significantly enriched in key genes involved in immune regulation, cytokine signalling, and cell adhesion (Svendsen et al., 2025). Studies have confirmed that the levels of validated DMPs are highly correlated with the pathological progression of RA, demonstrating their potential as key biomarkers for auxiliary diagnosis, prognostic prediction, and disease severity stratification (Mucientes et al., 2025). Studies indicate that methylation at the PCDH17 cg^03^865667 locus is a potential diagnostic marker for RA. Particularly in patients with seronegative RA, this marker demonstrates significant diagnostic advantages, helping to improve clinical detection rates and reduce rates of missed and misdiagnoses (Zhao F. et al., 2025). Another study indicates that DNA methylation levels at the HTR2A cg15692052 locus have the potential to serve as a diagnostic biomarker for RA and its clinical subtypes (Zhao J. et al., 2025). Furthermore, studies have confirmed that methylation levels at the AIM2 cg11003133 locus are highly correlated with the progression of RA. This epigenetic feature is highly consistent with its core biological function of mediating inflammasome activation, thereby inducing inflammatory cascades (Zhao et al., 2026).

Based on the association between LDLRAD4 and RA suggested by genome-wide association studies (GWAS), Jingjing Song et al. conducted a comparative study of 150 R A patients and 150 healthy controls and found that RA patients exhibited significantly lower methylation levels in LDLRAD4, with the differences primarily concentrated in the LDLRAD4-43 and LDLRAD4-44 regions (Song et al., 2025). The study found that the PDGFRB cg25613180 locus in patients with RA exhibited significant and highly specific hypomethylation, which could effectively distinguish them from healthy controls and patients with other rheumatic diseases. Given that methylation levels at this site are significantly negatively correlated with inflammatory markers (CRP, ESR), this suggests it plays a key role in the regulation of inflammation in RA and holds academic value for development as an RA-specific diagnostic biomarker (Shan et al., 2025). Other studies have confirmed that low methylation of the MMP9 promoter is a reliable marker for the diagnosis and prognostic assessment of RA, whilst the methylation level of the RUNX3 promoter has significant clinical value in monitoring disease activity. These epigenetic characteristics not only lay the molecular foundation for the precise diagnosis and stratification of RA, but also provide crucial support for the assessment of patient prognosis and personalised, refined management (Mohamadizad Moghadam et al., 2025). Further research has confirmed that the promoter regions of key disease-associated genes, such as IL-6, STAT3, MMP1 and TBX5, exhibit a significant pattern of low methylation. This epigenetic de-repression directly drives the overexpression of the associated genes and is highly correlated with systemic inflammation and the process of joint bone destruction in patients with RA. These findings collectively elucidate the epigenetic dysregulation characterised by the coexistence of global imbalances and site-specific variations in the pathological mechanisms of RA (Macias et al., 2026).

In summary, it is evident that abnormal DNA methylation patterns not only play a central role in the pathophysiology of RA, but also identify key targets for epigenetic therapies. Studies have confirmed that DNA methyltransferase inhibitors (DNMTis), by interfering with the catalytic activity of DNMTs, can precisely reverse hypermethylation of disease-associated genes, thereby restoring normal gene expression profiles. In clinical and research contexts, these inhibitors are typically categorised into two main groups—nucleoside analogues and non-nucleoside analogues—based on their chemical structure and pharmacological mechanisms (Huang et al., 2025). Furthermore, previous literature has indicated significant dysregulation in the expression of DNA methyltransferases during the progression of RA. Although initial clinical data suggest a general upregulation of DNMT1 transcription levels in patients, this expression profile exhibits significant dependence on the inflammatory microenvironment. Nakano et al. further elucidated this mechanism: under the stimulation of pro-inflammatory cytokines, DNMT1 and DNMT3a in FLS are, in fact, suppressed. In contrast, DNA demethylases in RA-specific immune cell subsets exhibit compensatory or pathological upregulation (Liu et al., 2011; Nakano et al., 2013; de Andres et al., 2015). DNA methylation modulators have emerged as a highly promising therapeutic approach in the treatment of RA. Studies indicate that these agents can reshape the epigenetic landscape via multiple pathways: For instance, azacitidine induces PBMCs to secrete the anti-inflammatory cytokine IL-10, whilst diminazene acetate, by targeting SSAT-1, restores DNMT1 expression and its mediated methylation modifications, thereby effectively curbing the invasive phenotype of FLS. These findings collectively confirm the feasibility and scientific value of DNA methylation pathways as novel therapeutic targets for RA (Danieli et al., 2024). Another study has found that in the collagen-induced arthritis (CIA) rat model, sinomenine effectively downregulates the expression of IL-6 by remodeling the methylation modification level of the IL-6 gene promoter, thereby exerting therapeutic effects on RA (Luo et al., 2026).

In summary, DNA methylation is not only central to the molecular mechanisms underlying RA, but also offers a highly promising potential avenue for precision clinical intervention. By specifically correcting abnormal methylation states in certain disease-associated genes, it may be possible to fundamentally reshape the synovial inflammatory microenvironment.

However, whilst the aforementioned studies have identified numerous differentially methylated sites (DMPs), it must be cautiously noted that current epigenetic research into RA suffers from a significant ‘correlation-to-causation gap’. Although existing epigenome-wide association studies (EWAS) have identified numerous sites, most lack validation of functional consistency, and their reproducibility across different ethnic cohorts remains a challenge. For example, whilst the methylation levels of loci such as PARP9 and PCDH17 have demonstrated diagnostic potential in experimental settings, their specificity as independent biomarkers—given the complex interference of multiple clinical factors—still requires validation in large-scale, prospective clinical cohorts. Future research should shift its focus from ‘identifying differential loci’ to ‘identifying functional epigenetic switches’, that is, determining which loci’s methylation changes truly drive pathological phenotypes, rather than merely serving as traces of metabolic and inflammatory stress in disease states.

### 3.2 Histone modifications

As another central pillar of epigenetic regulation, histone modifications play a pivotal role in shaping cellular phenotypes through the precise regulation of gene expression. Their epigenetic characteristics stem from highly complex PTMs, encompassing various forms such as acetylation, methylation, citrullination, phosphorylation, ubiquitination and glycosylation. These biochemical markers collectively drive the dynamic switching of chromatin between transcriptionally active states (euchromatin) and transcriptionally repressed states (heterochromatin) (Krishna Priya et al., 2025). Within the research landscape of histone modifications, acetylation and methylation have dominated; however, studies on RASFs—key pathological cells in RA—have revealed that histone citrullination is also a significant epigenetic feature (Araki and Mimura, 2016).

Histone acetylation has been identified as a key mechanism regulating the expression of the chemokine CCL2 (also known as MCP-1), the levels of which directly reflect disease activity in RA. The study further confirmed that, in an LPS-induced THP-1 cell model, histone acetylation in the CCL2 promoter region was significantly upregulated, providing strong evidence that this epigenetic modification is a key molecular event driving the surge in inflammatory cytokines (Badii et al., 2022). The dynamic balance between histone acetylation and deacetylation is a core epigenetic switch regulating gene transcription and cellular functional states. Histone acetyltransferases (HATs), using acetyl-CoA as a donor, attach an acetyl group to the ε-amino group of histone lysine residues, thereby neutralising the positive charge of histones and reducing their electrostatic affinity for DNA. This biochemical process induces a shift in chromatin conformation from compact to relaxed (chromatin decondensation), thereby enhancing the accessibility of transcription factors and activating gene expression. Conversely, histone deacetylases (HDACs) mediate the removal of acetyl groups, promoting the return of chromatin to a condensed state and leading to gene silencing (Vijaykrishnaraj et al., 2023). Although epigenetic interventions for RA remain in the early stages of clinical exploration, research into HDAC inhibitors has made substantial progress. Notably, the highly selective HDAC6 inhibitor CKD-506 has successfully completed a Phase II clinical trial (NCT04204603) in patients with refractory moderate-to-severe RA; Meanwhile, the pan-HDAC inhibitor ITF-2357 demonstrated excellent efficacy and safety in a Phase II trial for systemic juvenile idiopathic arthritis (sJIA) (NCT00570661), providing strong support for the clinical translation of this class of drugs (Huang et al., 2025). Secondly, methylation dysregulation in RA exhibits significant cell-specificity: although current evidence focuses primarily on CD4^+^ T cells—where low methylation of the TNF-α promoter and dysregulation of IL-21/34/RANKL expression are particularly characteristic—monocytes also display clear epigenetic heterogeneity (Pitaksalee et al., 2020).

Furthermore, lactylation, as an emerging PTM paradigm, forms a molecular bridge between cellular metabolism and signal transduction. Within this regulatory axis, lactic acid—accumulated as a result of abnormal glycolysis—transcends the traditional category of ‘metabolic waste’ and acts as a functional substrate to mediate modifications of both histones and non-histones, thereby enabling the epigenetic remodelling of cellular functions (Jiang et al., 2024). Specifically, histone lactylation achieves fine-tuned regulation of gene transcription by reshaping the epigenetic landscape, whilst non-histone lactylation modulates biological functions by inducing allosteric effects in proteins; the synergistic action of both jointly mediates the evolution of cellular pathophysiological states (Gao et al., 2024). Zhang et al. identified 28 lysine lactylation (Kla) sites in core histones from human and mouse cells, revealing the key role of this modification as a glucose metabolism sensor. Experiments confirmed that histone Kla levels correlate positively with intracellular lactate concentrations: lactate, whether accumulated through endogenous metabolism or supplied exogenously, directly drives Kla formation; conversely, pharmacologically blocking glycolysis or inhibiting lactate production effectively reverses this epigenetic process (Zhang et al., 2019).

Although research into histone modifications—one of the cornerstones of epigenetic regulation—in RA is currently largely confined to acetylation and methylation, they have already demonstrated broad potential for application. Future research should further explore their role in the pathogenesis of RA, with the aim of identifying highly specific biomarkers and therapeutic targets, thereby providing a theoretical foundation for the precise diagnosis and clinical management of RA.

### 3.3 Non-coding RNA

In the human genome, protein-coding sequences account for less than 3%, whilst the vast majority (approximately 76%–97%) of genomic sequences are transcribed into non-coding RNAs (ncRNAs), i.e., transcripts that lack the potential to be translated into functional proteins (Nemeth et al., 2024). Non-coding RNA (ncRNA) is widely recognised as a central regulatory hub for gene expression in the musculoskeletal system. Current research primarily covers three major functional lineages: miRNA, lncRNA and circRNA. Their molecular regulation exhibits a sophisticated network structure: miRNAs recognise and bind to target sites via seed sequences, mediating the suppression of mRNA translation or its degradation; simultaneously, lncRNAs and circRNAs exert a competitive endogenous RNA (ceRNA) effect, sequestering miRNAs through a ‘molecular sponge’ mechanism, thereby lifting the suppression of downstream target genes (Ali et al., 2021). The following text will provide a systematic discussion of the structural characteristics, biological functions and roles of miRNAs, lncRNAs and circRNAs in relevant pathological processes.

MicroRNAs (miRNAs) are endogenous non-coding RNAs approximately 22 nucleotides in length that primarily downregulate gene expression through post-transcriptional mechanisms. In recent years, the pathophysiological functions of miRNAs have attracted considerable attention; studies have shown that their abnormal expression is closely associated with the pathogenesis of malignant tumours and various autoimmune diseases, such as RA, systemic lupus erythematosus (SLE), Sjögren’s syndrome (SS) and systemic sclerosis (SSc) (Chang et al., 2022). Although genome-wide association studies (GWAS) have identified more than 100 genetic risk loci associated with RA, these known variants account for less than 40% of the heritability of RA, leaving a substantial ‘missing heritability’ to be elucidated. To further elucidate the underlying mechanisms, the team led by Shicheng Guo conducted a comprehensive genotyping analysis in a Chinese cohort comprising 1,607 patients and 1,580 controls. The study successfully identified six novel miRNA SNP loci significantly associated with RA risk, namely,: rs1414273 (miR-548ac), rs2620381 (miR-627), rs4285314 (miR-3135 b), rs28477407 (miR-4308), rs5997893 (miR-3928) and rs45596840 (miR-4482) (Guo et al., 2021). Meanwhile, within the pathophysiological mechanisms of RA, the regulatory role of miRNAs in FLS function has attracted considerable attention. On the one hand, Fang et al. demonstrated that miR-92a significantly inhibits the abnormal proliferation and migration of RA-FLS (Yu et al., 2018). On the other hand, research by Hao et al. indicated that miR-135b-5p is a key regulator of the inflammatory response in FLS (Hao et al., 2022). Other studies have shown that the overexpression of miR-106 b can effectively intervene in the malignant phenotype of RA-FLS, significantly inhibiting their proliferation, migration and invasive capabilities. By precisely downregulating relevant signalling pathways, miR-106 b not only curbs the cascade of synovial inflammation but also further alleviates damage to joint tissues. This significant antagonistic effect on the pathological progression of the synovium highlights the immense clinical value of miR-106 b as a potential therapeutic target for RA intervention (Liu et al., 2022). Furthermore, Luo et al. noted that the overexpression of miR-31 induces FLS proliferation and elevates levels of interleukin-1β (IL-1β) and tumour necrosis factor-α (TNF-α) (Luo et al., 2018). These pro-inflammatory factors, by activating protease activity and degrading the chondrocyte extracellular matrix, ultimately become key drivers of the pathological progression of RA and bone destruction (Law et al., 2021). Furthermore, studies on the underlying mechanisms have further revealed the dual role of miRNAs in the pathological progression of RA. On the one hand, miR-147b-3p has been shown to exert pro-inflammatory effects in RA and experimental arthritis models by specifically inhibiting ZNF148 (Jiang et al., 2021). On the other hand, research by Zhang et al. indicates that the absence of miR-150 downregulates IL-17 expression in T cells and joint tissues, thereby significantly alleviating the onset and pathological progression of arthritis in mice (Zhang and Zheng, 2024).

Lin-Lin Zhang et al. conducted an in-depth analysis of the association between genetic polymorphisms and disease based on genome-wide association studies (GWAS) data, combined with validation studies in independent samples. The results confirmed that the single nucleotide polymorphism (SNP) site rs2431697 in the miR-146a gene is significantly associated with susceptibility to RA (Zhang et al., 2021). Given the potential pathogenic effects of plasma exosomes in RA patients, the study further identified and confirmed that exosome-derived miR-144–3p and miR-30b-5p are significantly downregulated in patients. Notably, the low expression profile of these two miRNAs was highly correlated with RA disease activity indices. This finding provides strong evidence supporting their use as novel biomarkers for assessing disease progression and real-time monitoring (Lu J. et al., 2024). Jun Ma et al. analysed clinical samples from 32 pairs of RA patients and healthy controls, confirming that miR-345–3p is significantly downregulated in the serum of RA patients. Subsequent functional experiments demonstrated that upregulating miR-345–3p levels effectively inhibits the secretion of pro-inflammatory factors by RA-associated fibroblast-like synoviocytes (HFLS-RA) and can halt disease progression by antagonising abnormal cell proliferation and inducing apoptosis. These findings suggest that miR-345–3p holds promise as a novel biomarker for the clinical diagnosis of RA and a potential therapeutic target (Ma et al., 2023). Clinical data indicate that the expression levels of miRNA-146a in the peripheral blood of RA patients are significantly positively correlated with the inflammatory markers CRP and RF titres. Mechanistic studies revealed that intervention with oleuropein (OLEU) significantly downregulated TNF-α expression in PBMCs from RA patients, a process accompanied by a concurrent decrease in miRNA-146a levels. This finding suggests that OLEU may exert its anti-inflammatory effects by modulating miRNA-146a-mediated signalling pathways (Mahi et al., 2023).

In summary, miRNAs play a key role in the multidimensional pathological network of RA. Future research should focus on further exploring the regulatory potential of miRNAs and, through precise intervention in relevant molecular pathways, opening up new avenues for the early diagnosis and targeted treatment of RA.

Long non-coding RNAs (lncRNAs) are a class of transcripts that are longer than 200 nucleotides and do not encode proteins. As central components of cellular regulatory networks, lncRNAs mediate a wide range of biological functions; studies have shown that disruptions in their expression profiles (such as abnormal upregulation or silencing) and sequence variations are key molecular mechanisms driving the onset and progression of various diseases (Su et al., 2025). Most lncRNAs are transcribed by RNA polymerase II (RNAP II). Although they do not encode proteins, these molecules are classified by the scientific community as ‘mRNA-like lncRNAs’ due to structural features highly similar to those of mRNA, such as the 5′ cap structure and the 3′ poly(A) tail (Rinn and Chang, 2020). Long non-coding RNAs (lncRNAs), as a class of multifunctional regulatory factors, play a central role at both the transcriptional and post-transcriptional levels through complex interactive networks. Extensive research indicates that various lncRNAs, through distinct molecular mechanisms, profoundly mediate the pathological initiation and dynamic progression of RA (Ravaei et al., 2022).

NcRNAs are now widely recognised as key factors driving the pathological mechanisms of RA and influencing clinical prognosis. From a genetic perspective, the polymorphic site (rs2850711) of LNC00305 significantly increases the risk of developing RA; simultaneously, ncRNAs demonstrate pivotal value in regulating drug responsiveness, as differences in their expression profiles often mediate patients’ sensitivity to medication, thereby playing a significant role in the pathological progression of refractory RA (Yang et al., 2022). LncRNAs and circRNAs extensively exert a ‘miRNA molecular sponge’ effect, competitively binding to miRNAs to lift their suppression of target mRNAs, thereby precisely regulating the gene expression profiles of FLS, synovial cells and PBMCs. This ceRNA regulatory network profoundly mediates cellular proliferation, apoptosis, invasion and inflammatory cascades, providing key candidate targets for the precision diagnosis and treatment of RA (Han et al., 2022). Furthermore, lncRNAs exhibit multidimensional transcriptional repressive functions: in addition to acting as molecular sponges to disrupt the interaction between miRNAs and their binding proteins, they also mediate DNA epigenetic modifications, enabling fine-tuned regulation of gene expression at the chromatin level (Wang and Chang, 2011).

Long non-coding RNAs (lncRNAs) play a key regulatory role in the pathological progression of autoimmune diseases. Extensive evidence suggests that numerous lncRNAs are not only potential biomarkers for the diagnosis, prognosis assessment and monitoring of treatment response in RA, but also highly promising therapeutic targets (Elazazy et al., 2023). Their core mechanism lies in their deep involvement in the body’s complex inflammatory signalling pathways, precisely regulating the secretion of key pro-inflammatory factors such as TNF-α, IL-6, IL-8, IL-1β and type I interferon (IFN-I), thereby exerting a significant bidirectional regulatory effect on the progression of RA towards either exacerbation or remission (Wu et al., 2021). A wealth of research has confirmed that lncRNAs serve as central molecular hubs driving the development and lineage-specific differentiation of immune cells. Their regulatory effects span the entire process of immune system development: from the positive/negative selection of T lymphocytes within the thymus, to the maturation and development of B lymphocytes in the bone marrow, and even the functional specialisation of macrophages and dendritic cells (DCs) and the initiation of immune responses, lncRNAs play an indispensable and precise regulatory role (Chen et al., 2017).

Furthermore, the role of lncRNAs in regulating bone metabolic homeostasis has become a current research focus. Studies have demonstrated that lncRNAs trigger a cascade of matrix metalloproteinases (MMPs) and pro-inflammatory cytokines by mediating dysregulated interactions between RA-FLS and chondrocytes, thereby significantly accelerating the degradation of the cartilage matrix and the fibrotic transformation of synovial tissue. The interplay of these pathological processes ultimately leads to irreversible damage to joint structures, constituting the core driving force behind the pathological progression of RA (Miao et al., 2021). LncRNA is a key regulatory factor in the phenotypic transformation of RA-FLS towards proliferation and invasion. Studies have confirmed that the novel lncRNA LERFS is significantly downregulated in RA-FLS; its physiological function lies in recruiting the hnRNP Q protein to form a regulatory complex, thereby exerting a targeted inhibitory effect on the mRNAs of motility-related genes such as CDC42, RhoA and Rac1. Consequently, the homeostatic imbalance of the LERFS-hnRNP Q axis is regarded as one of the core molecular mechanisms driving abnormal proliferation and pathological invasion of the synovium in RA (Liu et al., 2023). Furthermore, LncRNA-IL17 R has been identified as a core regulatory factor driving the malignant proliferation of RA-FLS. Mechanistic studies reveal that LncRNA-IL17 R recruits and binds to EZH2 (Enhancer-like Homolog 2), inducing PRC2-complex-mediated epigenetic silencing, thereby downregulating the expression of the cyclin-dependent kinase inhibitors p21 (CDKN1A) and p16 (CDKN2A). Activation of this regulatory axis releases the brake on the cell cycle, significantly accelerating synovial cell proliferation during the pathological progression of RA (Ye et al., 2017). Recent research has revealed that lncRNA profoundly mediates bone erosion and bone destruction in RA by precisely regulating the autophagy pathway. This finding reveals a novel regulatory mechanism of lncRNA in bone metabolic disorders associated with RA (Lei et al., 2023).

Thanks to the widespread adoption of high-throughput microarray technology, breakthroughs have been made in the study of the complex regulatory networks underlying RA. Multiple studies have confirmed that long non-coding RNAs (lncRNAs) exhibit significant differential expression profiles in patients’ fibroblast-like synovial cells (FLSs), peripheral blood mononuclear cells (PBMCs), plasma and synovial tissue. This cross-tissue dysregulation not only reflects the pathological characteristics of RA but also reveals the central regulatory role of lncRNAs in driving the onset and progression of the disease (Fang et al., 2020). Through whole-transcriptome sequencing analysis of primary RA-FLSs, the study identified a total of 591 significantly differentially expressed lncRNAs (including 428 upregulated and 163 downregulated molecules). Functional enrichment analysis revealed that these differentially expressed molecules are central components of the inflammatory network within the synovial microenvironment, primarily driving the pathological progression of RA through the fine-tuning of Th17 cell polarisation, IL-6 receptor complex assembly, and the TGF-β signalling pathway (Shang et al., 2021). In the field of RA research, several validated lncRNAs (such as HOTAIR, GAS5 and HIX003209) are widely recognised as having significant potential for clinical translation. These molecules not only demonstrate high sensitivity and specificity in disease diagnosis but have also been identified as highly promising innovative therapeutic targets, providing a crucial basis for the realisation of precision medicine in RA (Wu et al., 2021).

In summary, lncRNAs play a key regulatory role in the early diagnosis and pathological progression of RA. In the future, further investigation into the gene regulatory mechanisms of lncRNAs and the development of targeted therapeutic strategies will be an important area of research for the clinical translation of RA.

Compared with linear RNA, circular RNA (circRNA) lacks a 5′cap and a 3′poly(A) tail due to its unique covalently closed-loop structure, which confers exceptional biological stability (Liu et al., 2023). It is precisely because of this characteristic that circRNA is considered a highly promising biomarker. Functionally, it not only acts as a ‘molecular sponge’ for miRNAs and RNA-binding proteins (RBPs), but also finely regulates the transcriptional elongation of RNA polymerase II (RNAP II) and the maturation processing of RNA (Han et al., 2022).

Recent in-depth studies have demonstrated that circRNA-mediated signalling pathways constitute a central mechanism regulating the pathological progression of autoimmune diseases. This not only establishes the status of circRNA as a core immunoregulatory factor but also provides a solid theoretical foundation for its translational research—particularly its development as a highly sensitive clinical biomarker (Lodde et al., 2020). A 2017 circular RNA microarray study revealed significant abnormalities in the circRNA expression profile in tissues from patients with RA. The analysis indicated that a total of 584 circRNAs were differentially expressed, with 255 upregulated and 329 downregulated (Zheng et al., 2017). Accumulating evidence suggests that extensive circRNA dysregulation exists in PBMCs from RA patients. Using a dual approach combining chip screening with RT-qPCR validation, Zheng et al. confirmed the significantly differential expression of multiple circRNAs, including hsa_circ_102,594, 103,334, 101,407, 104,593 and 104,194, in the pathological state of RA (Liu et al., 2023). Multiple studies collectively confirm that circRNAs and the complex molecular axes they mediate play a dual role in the pathological regulation of RA. Regarding pro-inflammatory and invasive mechanisms: Ouyang et al. found that circ_0005008 and circ_0005198 were significantly upregulated in the plasma of RA patients, with the latter potentially mediating the pathological evolution of RA-FLS through competitive binding to miR-4778–3p (Ouyang et al., 2021). Similarly, circ_0088194 may accelerate disease progression by negatively regulating the miR-766–3p/MMP2 axis; whilst circ_0088200 significantly induces the migration and invasion of RA-FLS by targeting miR-127–5p. These findings suggest that interfering with such pro-inflammatory signalling axes represents a potential strategy for mitigating the progression of RA (Cai et al., 2021). In terms of protective regulation: conversely, some circRNAs exhibit inhibitory effects on the pathological processes of RA. For example, circ-PTTG1IP is able to suppress the malignant phenotype of RA-FLS via the miR-671–5p/TLR4 axis (Chen et al., 2021). The protective circ_0008360 acts as a molecular sponge for miR-135b-5p, exerting its biological effects by downregulating HDAC4 levels (Hao et al., 2022).

Furthermore, regarding recent advances in clinical diagnosis and immune activation research: a recent independent validation cohort study comprising 160 samples (n = 78 R A vs. 82 H C) has further broadened the scope of research, identifying a total of 54 differentially expressed circRNAs. Among these, circRNA_0001412 and circRNA_0001566 were significantly upregulated in peripheral blood, demonstrating excellent diagnostic sensitivity and specificity. Bioinformatics predictions suggest that these two key molecules may drive abnormal T-cell activation by activating the PI3K-Akt signalling pathway, thereby mediating the pathological progression of RA from a new perspective of immune regulation (Li et al., 2026). Further evidence suggests that IL-6 and circ-DLGAP4 exert a potential synergistic regulatory role in the pathogenesis of RA. Not only are these two involved in the pathological progression of the disease, but they also hold promise as high-value biomarkers, offering new avenues for research into the clinical screening and auxiliary diagnosis of RA (Mahdi et al., 2026).

In 2011, the competitive endogenous RNA (ceRNA) hypothesis proposed by Salmena et al. provided a core framework for understanding the function of non-coding RNAs. This hypothesis elucidates how circRNAs and lncRNAs, through complementary binding sites abundant in their sequences, act as molecular sponges for miRNAs, thereby relieving miRNA-mediated translational repression or degradation of target mRNAs via a ‘sequestration’ effect (Wen et al., 2023). At the transcriptional and post-transcriptional levels, this circRNA-miRNA-mRNA regulatory network exhibits extreme complexity and serves as a key mechanism for the precise regulation of gene expression and the mediation of various disease pathophysiological processes (Han et al., 2022). In the pathological progression of RA, multiple ceRNA axes have been shown to exert distinct regulatory functions. Anti-inflammatory and protective effects: Luo et al. found that the circMAPK9/miR-140–3p/PPM1A axis exerts a protective effect in RA-FLSs. Highly expressed circMAPK9 upregulates PPM1A by sponging miR-140–3p, thereby inhibiting inflammation, proliferation and migration in synovial cells and inducing their apoptosis. Conversely, knockdown of circMAPK9 exacerbates the pathological process by attenuating this sponging effect (Luo et al., 2021). Pro-inflammatory and pathogenic effects: In contrast, Yang et al. demonstrated that circRNA_09505 is significantly overexpressed in PBMCs from RA patients, where it lifts the inhibition of AKT1 by competitively binding to miR-6089. This process activates the downstream IκBα/NF-κB signalling pathway, inducing the massive secretion of pro-inflammatory factors and significantly exacerbating the progression of experimental arthritis (CIA) (Yang et al., 2020). Clinical studies based on high-throughput sequencing have further validated the complexity of ceRNA networks. Through the analysis of 25 pairs of samples from RA patients and healthy controls, the study identified a differential expression profile comprising 1,366 mRNAs, 47 circRNAs and 223 miRNAs. Among these, hsa_circ_0092125 and its host gene G6PD were simultaneously and significantly upregulated in RA samples. Network analysis revealed that this axis occupies a central position in the immune-inflammatory pathway, suggesting that hsa_circ_0092125/G6PD is not only a highly promising clinical diagnostic biomarker but also provides a new direction for molecularly targeted therapy in RA (Golestanifar et al., 2025).

In summary, circRNAs are not only regarded as promising biomarkers for the early diagnosis of RA, but are also deeply involved in the pathological progression of the disease. In the future, further mapping of the differential expression profiles of circRNAs and the exploration of targeted intervention strategies will be key areas of development in advancing RA diagnosis and treatment research.

## 4 Interference between key metabolic products and epigenetics in RA

**FIGURE 4:**
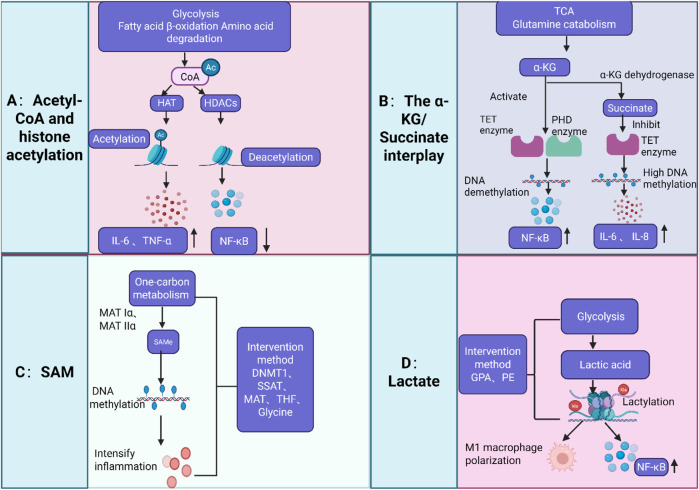
Epigenetic reprogramming mediated by metabolic intermediates in RA and intervention strategies. This figure elaborates the regulatory mechanisms of four key metabolites in the epigenetic pathology of RA: **(A)** Acetyl-CoA: It describes the metabolic products of glycolysis, fatty acid β-oxidation, and amino acid degradation, such as acetyl-CoA, which are added with acetyl groups by histone acetyltransferase (HAT) to undergo histone acetylation, promoting the transcriptional activation of genes and the release of inflammatory factors (IL-6, TNF-α). Conversely, acetyl-CoA undergoes deacetylation by histone deacetylase (HDAC) by removing this group, inhibiting gene expression and reducing the release of NF-κB. **(B)** Interaction between α-ketoglutarate (α-KG) and succinate: It describes the metabolic products of the TCA cycle and glutamine decomposition, α-ketoglutarate, which activate TET and prolyl hydroxylase (PHD) to promote DNA demethylation, blocking the classical NF-kB inflammatory signaling pathway, thereby synergistically inhibiting the pro-inflammatory response. At the same time, α-KG is catalyzed by α-KG dehydrogenase to decarboxylate and generate succinate, promoting DNA hypermethylation by inhibiting the TET enzyme and promoting the secretion of inflammatory factors such as IL-6 and IL-8. **(C)** S-adenosylmethionine (SAM): It describes the product of one-carbon metabolism, SAM, which exacerbates the inflammatory response and its targeted intervention strategies. **(D)** Lactic acid: It describes the metabolic product of glycolysis, lactic acid, which mediates lactylation modification to promote M1 macrophage polarization and activate the classical NF-kB inflammatory signaling pathway and its targeted intervention strategies.

RA is a chronic systemic autoimmune disease characterised by persistent synovitis and progressive destruction of peripheral joints and soft tissues (Di Matteo et al., 2023). Cellular metabolic reprogramming plays a central regulatory role in its complex pathogenesis (Jia et al., 2023). More importantly, as a key hub for cells to sense environmental changes, the metabolic state is deeply involved in the remodelling of the epigenetic landscape: intermediate metabolites can precisely regulate chromatin conformation dynamics and downstream gene expression by mediating PTMs of chromatin (Sun et al., 2022). Further studies have revealed that the metabolites acetyl-CoA, α-ketoglutarate, succinate, S-adenosylmethionine and lactate play a key regulatory role in the pathological progression of inflammatory diseases, primarily by reshaping the epigenetic landscape of cells, thereby profoundly influencing clinical outcomes (Zhang et al., 2019; Gyanwali et al., 2022; Sun et al., 2022; Huang et al., 2024; Wang et al., 2025) (Figure 4). Consequently, a thorough elucidation of the pathways leading to the formation of RA intermediate metabolites, together with an understanding of the epigenetic modification mechanisms they mediate, their pathogenic roles and potential therapeutic interventions (Table 1), will provide a solid theoretical foundation for the development of novel RA treatment strategies.

**TABLE 1:** The epimetabolic crosstalk in RA: mechanisms and therapeutic potential.

Metabolic intermediates	Associated epigenetic modifications	Pathogenic effects	Potential therapeutic interventions	References
Acetyl-CoA	H3K9ac, H3K27ac	Activate the transcription of inflammatory genes (IL-6, TNF-α) and maintain the invasive nature of FLS	Curcumin,CKD-506, Wutou Decoction (WTD)	Wada et al. (2014) Huang et al. (2025) Wen J. et al. (2025)
Succinate	High DNA/histone methylation (inhibite TET/KDM)	Block the expression of the anti-inflammatory gene (SFRP2)	Succinate dehydrogenase (SDH) targeted regulation, 5-azadC	Miao et al. (2018)
α-Ketoglutarate (α-KG)	H3K27me3 reduction	Drive DNA demethylation and relieve gene silencing, and promote the expression of related genes	α-KG supplementation	Liu et al. (2017)
Lactate	H3K18La, H3K56La	Promote the activation of the cGAS-STING pathway and induce the shift of macrophages towards the M1 phenotype	Luteolin (LUT),Gastrodin (GAS),Parishin E (PE)	Jiang et al. (2026) Dai et al. (2025) Liu X. et al. (2025)
S-adenosylmethionine (SAM)	Whole-genome DNA/histone methylation	Lead to hypomethylation of the promoters of pro-inflammatory factors, which triggers a pathological inflammatory cascade	SAM supplementation, itaconate, and folic acid	Frank-Bertoncelj and Gay (2014) Tada and Kono (2025) Meng et al. (2026)

### 4.1 Acetyl-CoA and histone acetylation

Acetyl-CoA is a central metabolic hub that deeply integrates the metabolism of carbohydrates, lipids and amino acids (such as glutamine) with cellular signal transduction (Wang et al., 2025). Within the cell, acetyl-CoA is primarily derived from glucose breakdown (glycolysis), fatty acid β-oxidation and amino acid degradation pathways (Xu et al., 2026). Functionally, it serves not only as a key precursor substrate for the *de novo* synthesis of fatty acids and cholesterol, but also as an indispensable acetyl group donor for protein acetylation (Wang et al., 2025). Intracellular acetyl-CoA production is primarily mediated by ATP-citrate lyase (ACLY) and acetyl-CoA synthase 2 (ACSS2), which convert citrate and acetate, respectively (Yang et al., 2025). In particular, ACLY-mediated acetyl-CoA production within the nucleus serves as a core mechanism for maintaining histone acetylation and epigenetic homeostasis in pluripotent stem cells by providing the essential acetyl groups required for chromatin modification (Li et al., 2018).

Epigenetic modifications of histones refer to chemical covalent modifications occurring on the N-terminal amino acid residues of histones. Without altering the DNA sequence, these modifications precisely drive gene transcription regulation by reshaping the three-dimensional structure of chromosomes and modulating chromatin accessibility (Su et al., 2025). Among these, histone acetylation and deacetylation constitute a classic dynamic equilibrium process. Histone acetylation and deacetylation are dynamically catalysed by HATs and HDACs, respectively. The former activates DNA transcription by adding an acetyl group, whilst the latter inhibits or regulates gene expression by removing this group; together, they constitute the core regulatory mechanism of gene transcription (Pai et al., 2024). Based on subcellular localisation and structural characteristics, HATs are primarily classified into three major classical families: the GNAT family, the MYST family, and the p300/CBP family. Furthermore, HATs, acting as ‘writers’ of epigenetic marks, neutralise the positive charge of histones by catalysing the acetylation of lysine residues at the histone tails, thereby weakening the electrostatic attraction between histones and DNA. This dissociation process promotes a shift in chromatin structure from compact to relaxed (i.e., chromatinisation), greatly increasing DNA accessibility and thereby facilitating the efficient binding of transcription factors to target sequences (Xu et al., 2026).

Conversely, curcumin, acting as a histone acetyltransferase (HAT) inhibitor, can block the transcriptional activation of this gene by significantly downregulating H3 acetylation (H3ac) levels in the IL-6 promoter region, thereby effectively inhibiting the expression of IL-6 mRNA and the secretion of its protein in RASFs (Wada et al., 2014). Furthermore, HDACs, acting as epigenetic ‘erasers’, restore the compact structure of chromatin by removing acetyl groups from histones. Based on cofactor dependence and sequence homology, the HDAC family is primarily divided into four major classes: classical HDACs (comprising classes I, II and IV) and class III HDACs (i.e., the Sirtuin family) (Xu et al., 2026). Research findings over the past 2 decades have established the important role of histone deacetylase inhibitors (HDACi) in the treatment of RA. By blocking HDAC activity, these inhibitors can significantly suppress pro-inflammatory cytokine cascades in the pathological microenvironment of RA, thereby effectively delaying or reversing disease progression (Pai et al., 2024).

In the pathological progression of inflammatory bone diseases, the functional imbalance between HATs and HDACs is the core mechanism driving the abnormal release of pro-inflammatory factors. Specifically, HATs catalyse the acetylation of histones in the NF-κB p65 subunit or the promoter regions of its target genes, thereby significantly activating the transcription of factors such as IL-6 and TNF-α, which in turn exacerbates the inflammatory microenvironment within the joint (Hu et al., 2021). Concurrently, the abnormally high expression of HDACs silences the transcription of anti-inflammatory genes through deacetylation, serving as a crucial component in sustaining the inflammatory cascade. Existing studies have confirmed that broad-spectrum HDAC inhibitors (such as TSA and SAHA) can effectively block the transcriptional activity of NF-κB in mouse models, downregulate levels of inflammatory cytokines, and successfully restore the body’s immune homeostasis (Weiss et al., 2020).

Recent studies have revealed a complex network of mutual regulation between cellular metabolism and histone acetylation in macrophages. As a key acetyl donor, the metabolic product acetyl-CoA is deeply involved in HAT-mediated enzymatic reactions, thereby directly coupling the cell’s energy metabolic state to the epigenetic landscape (Xu et al., 2026). Specifically, pro-inflammatory M1 macrophages significantly elevate acetyl-CoA levels by enhancing glycolytic flux, thereby driving histone acetylation in the promoter regions of inflammation-related genes; in contrast, oxidative phosphorylation-dependent M2 macrophages exhibit distinct histone acetylation profiles that specifically promote the expression of anti-inflammatory genes (Zhang and Jagannath, 2025). This metabolic-epigenetic coupling mechanism plays a key role in musculoskeletal disorders. Clinical evidence suggests that systemic metabolic disorders, such as diabetes, exacerbate pathological inflammation by reshaping the epigenetic landscape, thereby impairing skeletal regenerative potential and delaying healing (Xu et al., 2026). Given the central role of histone deacetylase 6 (HDAC6) in inducing the expression of pro-inflammatory factors in macrophages, the development of inhibitors targeting HDACs (such as HDAC6) has emerged as a potential strategy for treating macrophage-associated chronic immuno-inflammatory diseases such as RA and atherosclerosis (Yang H. M. et al., 2024).

In summary, the key metabolite acetyl-CoA is not only deeply involved in the pathological process of RA, but also acts as an indispensable acetyl donor that directly mediates protein acetylation. Consequently, targeting the regulation of acetyl-CoA production at its source, or intervening in epigenetic modifications through the coordinated regulation of HATs and HDACs, represents an important direction for future research into the treatment of RA.

### 4.2 The interplay between α-ketoglutarate (α-KG) and succinate

The TCA cycle is a central hub for cellular energy metabolism and biosynthesis (Eniafe and Jiang, 2021). α-Ketoglutarate is involved in several key metabolic pathways, including glutamine catabolism and the TCA cycle. In particular, glutamine can be sequentially converted into glutamate and α-ketoglutarate via the classical catabolic pathway (Vasconcelos et al., 2024). α-KG is a central intermediate in numerous metabolic and signalling pathways. It not only plays a key role in energy metabolism networks such as the carbohydrate-based tricarboxylic acid (TCA) cycle (Wu et al., 2025), but also possesses multiple physiological functions, including antioxidant activity and regulation of nitrogen-ammonia balance, and is deeply involved in the regulation of epigenetic modifications and immune function (Gyanwali et al., 2022).

As a central hub in the mammalian metabolic network,α-KG exhibits multiple core biological functions, including linking intracellular carbon and nitrogen metabolism, regulating epigenetics, and intervening in skeletal system diseases. In terms of biochemical metabolism,α-KG is extensively involved in multiple biosynthetic and catabolic pathways: Firstly, in the TCA cycle, α-KG undergoes decarboxylation catalysed by α-ketoglutarate dehydrogenase to produce succinate, carbon dioxide and water, and is ultimately completely oxidised to generate energy; secondly, in amino acid metabolism, it combines with free ammonia, catalysed by glutamate dehydrogenase, to form glutamate, which serves as a precursor for the further synthesis of glutamine and glutathione (Singh et al., 2017; Cheng et al., 2024). Thirdly, it can also serve as a carbon skeleton for glucose production via gluconeogenesis, or participate in the synthesis of glycerol and fatty acids (Singh et al., 2017). In terms of cellular signalling and immune regulation, α-KG, as a metabolic analogue of glutamine, effectively inhibits protein degradation and promotes nitrogen retention; simultaneously, it not only acts as a reactive oxygen species (ROS) scavenger to counteract oxidative stress, but also functions as a signalling molecule to regulate G protein activity, thereby playing a key role in immune modulation and the control of cell growth (Mangal et al., 2021). In the context of epigenetics and disease intervention, α-KG serves as a key bridge linking cellular metabolic pathways and epigenetic regulation. On the one hand, it promotes histone acetylation by regulating the homeostasis of acetyl donors; on the other hand, it indirectly inhibits the differentiation of osteoclasts (OCs) by modulating the activity of H3K9 demethylases (Xu et al., 2026). Based on this unique metabolic-epigenetic coupling mechanism, α-KG has demonstrated broad potential as a therapeutic target for various skeletal system diseases, including osteoporosis, osteoarthritis and RA (Wu et al., 2025).

Transcriptional regulation in eukaryotes is highly dependent on methylation modifications of nucleosome histones, and this dynamic balance is finely tuned by histone demethylases containing JmjC domains (JMJs/KDMs). Recent research has revealed that α-ketoglutarate dehydrogenase (KGDH), a key enzyme in the TCA cycle, is capable of nuclear translocation and, through direct interaction with various JMJs, locally regulates their demethylation activity (Huang et al., 2023). In this nuclear metabolic-epigenetic coupling mechanism, α-KG plays a central role as the core cofactor of the α-KG-dependent dioxygenase family. This family includes TET DNA hydroxylases and JMJD histone lysine demethylases. Taking JMJD as an example, it utilises α-KG and oxygen as co-substrates to catalyse the demethylation of histone lysine residues (Randhawa and Pålsson-McDermott, 2025). From a pathophysiological perspective, α-KG-mediated epigenetic remodelling acts as a key switch for reversing the inflammatory response: under normal conditions, excessive histone methylation (such as H3K27me3) promotes chromatin compaction, thereby suppressing the expression of anti-inflammatory genes such as Arg1. α-KG binds to and activates JMJD proteins, removing the ‘shackles’ of methylation and opening the DNA conformation, thereby restoring the transcription of anti-inflammatory genes; simultaneously, α-KG acts as a cofactor to activate TET enzymes, driving DNA demethylation (generating the 5-hydroxymethylcytosine 5 hmC intermediate marker), lifting gene silencing and promoting the expression of relevant genes (Liu et al., 2017). In addition to epigenetic modifications, α-KG can also activate prolyl hydroxylase (PHD), which blocks the classical NF-κB inflammatory signalling pathway by modifying the IKKβ protein, thereby synergistically inhibiting pro-inflammatory responses (Liu et al., 2017).

As a key metabolite in the TCA cycle, succinate is produced from α-ketoglutarate via a two-step reaction: first, α-ketoglutarate is oxidised and decarboxylated by α-ketoglutarate dehydrogenase to form succinyl-CoA, which is then converted to succinate by succinyl-CoA synthetase. This process constitutes substrate-level phosphorylation, which directly couples to the production of one molecule of GTP (or ATP in some organisms) (Martínez-Reyes and Chandel, 2020). Succinate occupies a pivotal position in cellular metabolism: not only does it act as a substrate for succinate dehydrogenase (SDH) in catalytic reactions, but it also directly introduces electrons into the electron transport chain (ETC.) via this enzymatic reaction (Huang et al., 2024). For a long time, succinate was widely regarded as merely an energy metabolism intermediate in the TCA cycle. However, recent research has challenged this traditional view, confirming that it is also a key regulator of inflammation (Huang et al., 2024). The dual role of intracellular succinate as a key pro-inflammatory mediator is attracting widespread attention and in-depth research within the field of immunometabolism (Murphy and O'Neill, 2018). When cellular metabolism is disrupted, succinate accumulates in large quantities within the cell and leaks out. Within cells, it activates a specific protein (HIF-1α) to exacerbate inflammation. Outside cells, it acts like a signal light, binding to immune cells to directly orchestrate the immune response. This abnormal accumulation of succinate is a key factor in the development of various inflammatory diseases, including RA (Huang et al., 2024).

Epigenetic studies have further elucidated the regulatory mechanisms of metabolic abnormalities in inflammatory diseases. Xiao et al. found that knockdown of succinate dehydrogenase (SDH) leads to a significant accumulation of intracellular succinate. As a structural analogue of α-KG, accumulated succinate competitively inhibits α-KG-dependent dioxygenases (such as the TET and KDM families), leading to a significant increase in global histone methylation levels and a marked reduction in the DNA demethylation product 5 hmC due to the inhibition of TET1/2 activity (Xiao et al., 2012). This DNA hypermethylation-mediated gene silencing is a key mechanism driving the progression of RA. In a rat model of RA, DNMT1 directly targets and methylates the promoter of the anti-inflammatory gene SFRP2, leading to its silencing, thereby lifting the inhibition of the canonical Wnt signalling pathway and promoting the pathogenic activation of FLS. Experiments confirmed that knocking down DNMT1 or using the demethylating agent 5-azadC restored SFRP2 expression, thereby significantly inhibiting FLS proliferation and the secretion of pro-inflammatory factors such as IL-6 and IL-8 (Miao et al., 2018).

In summary,α-KG and succinate—key metabolites of TCA cycle—play a pivotal bridging role in regulating the inflammatory response in joints. In the future, elucidating the immunometabolic regulatory mechanisms mediated by these two compounds and using this understanding to develop precision-targeted therapeutic strategies will be a key focus of translational medical research into arthritis.

However, a thorough analysis of the current literature reveals significant heterogeneity in the strength of evidence supporting the ‘metabolite-driven epigenetic modification’ model. Much of the existing evidence stems from intervention models using high concentrations of exogenous metabolites or specific gene-knockout experiments; there is still a lack of direct *in vivo* imaging evidence to support whether metabolic fluctuations at physiological concentrations within the body are sufficient to induce chromatin structural remodelling. Particularly within the synovial microenvironment of RA patients, local concentrations of metabolites (such as succinate and lactate) are highly dynamic and subject to multiple constraints, including hypoxia, nutrient deprivation and the formation of vascular pseudopodia. Current research tends to describe a ‘unidirectional causal’ relationship, yet the pathological process of RA is more likely to be a complex feedback loop. We must therefore carefully assess whether metabolites act as precise ‘signal transducers’ or merely as ‘metabolic by-products’ following inflammatory cascades. The key to resolving this debate lies in developing single-cell metabolic analysis techniques capable of real-time monitoring of the coupling between local metabolic flux in synovial tissue and specific epigenetic modifications, thereby overcoming the experimental limitations associated with interventions relying on exogenous additions.

### 4.3 S-adenosylmethionine (SAM) and methylation

The synergistic operation of the methionine cycle and the folate cycle forms the core of cellular one-carbon metabolism. The key metabolite generated by this process, S-adenosylmethionine (SAMe), is the most important universal methyl donor in mammals and plays a dominant role in driving epigenetic methylation modifications of DNA and histones (Sun et al., 2022). The intracellular synthesis of SAM is an ATP-dependent, energy-coupled process. As the central rate-limiting step of this cycle, methionine undergoes a condensation reaction with ATP, catalysed by the rate-limiting enzymes MAT Iα or MAT IIα, to form SAM (Sun et al., 2022). Subsequently, after releasing a methyl group to the substrate, SAM is converted to S-adenosylhomocysteine (SAH), which is then rapidly converted to homocysteine (Hcy) by deadenylation via SAH hydrolase (SAHH). To maintain the continuity of the cycle, Hcy accepts a one-carbon unit from 5-methyltetrahydrofolate (5-mTHF) in the folate cycle and is remethylated to regenerate methionine, thereby completing the metabolic cycle of the methionine cycle (Locasale, 2013).

As the central donor for various intracellular methylation modifications, the intracellular concentration of SAM is precisely regulated by multiple feedback and homeostatic mechanisms to maintain it at an optimal physiological level (Xing and Tu, 2025). The SAM/SAH ratio is used as a marker of methylation and is a key indicator of a cell’s methylation capacity (Aso et al., 2023). Under methionine-limited conditions, a dynamic imbalance occurs in the levels of SAM and S-adenosylhomocysteine (SAH) within cells, as well as in their ratio (SAM/SAH), leading to a significant downregulation of H3K4me3 modification levels. This alteration in metabolic signalling further regulates the expression profile of methylation-related enzymes via a feedback mechanism, thereby facilitating the remodelling of the epigenetic landscape from metabolite abundance (Mentch et al., 2015). Imbalances in DNA methylation homeostasis are closely associated with the pathological progression of autoimmune diseases. Studies have shown that epigenetic modifications centred on DNA methyltransferases (DNMTs) play a key role in the pathogenesis of RA, whilst intracellular levels of methylation donors and products (SAM and SAH) directly determine the body’s methylation flux and epigenetic state (Tao et al., 2008). Within the pathological microenvironment of RA, FLS/RASF exhibit unique metabolic and epigenetic characteristics: on the one hand, pro-inflammatory cytokines exacerbate the downregulation of DNMT1 during FLS proliferation, leading to global hypomethylation of CpG sites; on the other hand, enhanced intracellular polyamine cycling and activation of SSAT activity lead to abnormal depletion of the methyl donor SAM (Frank-Bertoncelj and Gay, 2014). This anomalous coexistence of ‘high levels of SAM consumption/uptake’ and ‘genome-wide DNA hypomethylation’ suggests the distinctiveness of RA FLS in terms of methylation utilisation efficiency or regulatory pathways (Karouzakis et al., 2012). Several recent studies have shown that a relative scarcity of the methyl donor SAM in the RA microenvironment leads to global DNA hypomethylation in FLS. This widespread epigenetic alteration disrupts the native gene expression profile, thereby accelerating the pathological progression of RA (Ling et al., 2022). Consequently, exogenous supplementation of SAM combined with the inhibition of SSAT activity holds promise as an innovative epigenetic intervention strategy for RA FLS (Frank-Bertoncelj and Gay, 2014).

Furthermore, in the classical biochemical pathway, SAM is derived from methionine through a reaction catalysed by methionine adenosyltransferase (MAT). However, recent studies have revealed that this synthetic process exhibits specific metabolic regulation in Th17 cells: the enzymatic activity of MAT is significantly inhibited by the endogenous immunometabolite itaconate (Aso et al., 2023). As a key immunometabolic regulator, itaconate profoundly reshapes the metabolic and epigenetic landscape of T cells by reducing 2-hydroxyglutarate (2-HG) levels and downregulating the SAM/SAH methylation index. This dual regulatory effect effectively suppresses the pathogenic differentiation of Th17 cells whilst enhancing the stable expression of Foxp3 in Treg cells. Preclinical studies have further confirmed that itaconate demonstrates significant therapeutic potential in mitigating the pathological progression of various autoimmune diseases, including RA, systemic lupus erythematosus (SLE) and experimental autoimmune encephalomyelitis (EAE) (Tada and Kono, 2025).

Glycine is an important carbon source for one-carbon metabolism and indirectly participates in the regulation of methylation by promoting the synthesis of SAM (Shyh-Chang et al., 2013). In the pathophysiology of RA, glycine does not exert its antioxidant effects as a substrate for glutathione (GSH) synthesis, but rather acts as a one-carbon unit donor, mediating epigenetic remodelling via SAM-dependent pathways (Ling et al., 2022). *In vivo* experiments confirmed that glycine treatment significantly elevates intracellular SAM levels, accompanied by downregulation of GPX4, a key enzyme involved in anti-apoptosis (Zhang et al., 2020). As the GPX4 upstream promoter region exhibits low methylation levels under basal conditions, this suggests that glycine suppresses GPX4 transcription at the epigenetic level by enhancing SAM-mediated promoter-specific hypermethylation of GPX4, thereby inducing and amplifying the ferroptosis process in RA synovial cells (Zhang et al., 2020; Ling et al., 2022).

Tetrahydrofolate (THF) and its predominant serum form, 5-methyl-THF, are key carbon carriers in one-carbon metabolism, directly mediating the conversion of homocysteine into the universal methyl donor SAM to maintain the homeostasis of genome-wide DNA methylation (Navarro-Rodríguez et al., 2026). Inadequate dietary folate intake or MTHFR gene polymorphisms can significantly limit the supply of SAM, leading to global DNA demethylation, thereby compromising genomic stability and inducing gene expression dysregulation (Sun et al., 2022; Navarro-Rodríguez et al., 2026). It is worth noting that methotrexate (MTX), a first-line treatment for RA and an inhibitor of dihydrofolate reductase (DHFR), is highly likely to cause folate depletion in patients whilst alleviating joint symptoms. Consequently, the combination of folic acid and MTX is widely adopted in clinical practice, aiming to counteract the metabolic side effects caused by MTX through exogenous folic acid supplementation and thereby protect the body’s epigenetic homeostasis (Meng et al., 2026).

In summary, the central carbon source (glycine), the one-carbon unit carrier (tetrahydrofolate, THF) and its key metabolite (S-adenosylmethionine, SAM) in one-carbon metabolism play a pivotal bridging role in the pathophysiological mechanisms of RA. Among these, the SAM/THF ratio, as a core quantitative indicator for assessing cellular epigenetic methylation capacity, directly determines the remodelling of gene expression profiles in RA synovial tissue and immune cells. Consequently, the precise regulation of the metabolic flux of glycine, THF and SAM, and the subsequent intervention in SAM-mediated abnormal methylation modifications, has become a crucial breakthrough for the future development of innovative metabolic-targeted therapeutic strategies for RA.

### 4.4 Lactate and histone lactation

Traditionally, lactate (LA)—derived from pyruvate—has been regarded merely as an energy source or a metabolic by-product. However, recent evidence suggests that lactate also acts as a key epigenetic signalling molecule, directly activating chromatin transcription of specific target genes by mediating a novel ‘lactylation’ modification of histone lysine residues (Zhang et al., 2019). Intracellular metabolites frequently act as signalling molecules, regulating gene expression by mediating PTMs of proteins such as histones. Based on this mechanism, Zhang et al. have recently identified a novel histone modification driven by LA, which they have termed ‘lactylation’ (Izzo and Wellen, 2019; Zhang et al., 2019). Studies indicate that lactylation plays a key role in the regulation of bone metabolism: on the one hand, it directly regulates osteoblast differentiation and osteoclast activity; on the other hand, it mediates complex “immune-bone axis” crosstalk by modulating the functions of immune cells, such as T-cell and macrophage polarisation. This multidimensional regulatory mechanism not only effectively maintains bone homeostasis in the microenvironment but also profoundly influences local inflammatory progression and tissue repair (Ye et al., 2025).

The microenvironmental characteristics of the synovium in RA—characterised by hypoxia, high inflammation and nutrient deprivation—induce a typical ‘Warburg effect’ in synovial cells (i.e., a metabolic shift from oxidative phosphorylation to glycolysis). High-efficiency glycolysis not only provides ample energy and biosynthetic precursors for the malignant proliferation of pathogenic cells, but the abnormal accumulation of its metabolic by-product, lactate, in the microenvironment also plays a central regulatory role: by reshaping the local environment, activating specific signalling pathways, and mediating ‘lactylation’ modifications of histones and non-histones, it significantly enhances the pathogenic functions of synovial cells (Gan et al., 2024). Research findings indicate that lactic acid (LA) is significantly enriched in the synovial fluid of patients with RA and has become a reliable biomarker for identifying inflammatory joint lesions (Yang H. M. et al., 2024). Furthermore, abnormally accumulated LA can specifically act on various key immune and stromal cells, including FLS, macrophages and T cells, thereby acting as a cascade amplifier to drive local inflammatory responses in the joint (Gan et al., 2024). Single-cell sequencing analysis indicates that lactation scores are significantly elevated in patients with RA, and are positively correlated with the extent of immune cell infiltration and the expression levels of immune checkpoint molecules. Concurrently, key pro-lactation genes such as NDUFB3, NGLY1 and SLC25A4 are highly expressed in RA. These findings suggest that these characteristic genes and the lactic acid-related phenotypes they mediate hold promise as potential clinical biomarkers for RA (Fu et al., 2024). The study indicates that histone lactic acid modifications (particularly H3K18La) are significantly elevated in the synovial tissue of RA patients. In a CIA model, inhibition of H3K18La effectively blocked the TNF-α-induced pathogenic phenotype in FLS and alleviated joint symptoms; conversely, its accumulation exacerbated pathological responses by synergistically regulating chemokine signalling and TRP channel-related inflammatory mediators. Mechanistic investigations revealed that SIRT3, the key deacetylase of H3K18La, is downregulated in PBMCs from RA patients; this downregulation of SIRT3 directly lifts the repression of acetylation, leading to the massive accumulation of H3K18La in macrophages and FLS and the abnormal activation of FLS, thereby accelerating the progression of RA (Zheng et al., 2025).

As key multifunctional immune effector cells, macrophages not only eliminate pathogens, tumour cells and senescent cells through phagocytosis, but are also deeply involved in the remodelling and repair of damaged tissues. Consequently, macrophages play a crucial defensive role in the body’s response to infection, injury and tumours, as well as in maintaining immune homeostasis (Wang et al., 2019). In the regulation of macrophages, M1 and M2 types play diametrically opposed roles in disease progression: the former exacerbates joint destruction and bone erosion by releasing pro-inflammatory factors, whilst the latter secretes anti-inflammatory mediators to alleviate inflammation and promote tissue repair (Kulakova et al., 2024).

However, within the pathological microenvironment of RA, multiple pathogenic factors disrupt this inherent immune homeostasis, driving abnormal macrophage polarisation towards the pro-inflammatory M1 phenotype (Zheng et al., 2024). This M1/M2 imbalance, characterised by a predominance of M1 macrophages, not only persistently amplifies local synovial inflammation but also constitutes a core pathogenic mechanism driving the progression of RA (Culemann et al., 2019). In the RA microenvironment, lactate significantly amplifies local inflammatory responses through a cascade of effects by driving metabolic reprogramming of synovial cells and immune cells. Crucially, lactate exerts a unique dual-track effect on macrophage polarisation: under specific conditions, it can exacerbate pro-inflammatory pathology, whilst also inducing macrophage polarisation towards the anti-inflammatory/pro-repair M2 phenotype, thereby playing a multidimensional regulatory role in RA disease progression and tissue remodelling (Chen et al., 2026). In synovial macrophages from RA patients and mouse models, levels of dsDNA, LA and cGAS protein are abnormally elevated. Mechanistic studies indicate that accumulated lactate mediates the lactylation of the cGAS protein, thereby inhibiting its degradation and leading to abnormal intracellular accumulation of cGAS. This stably expressed cGAS continuously activates the downstream cGAS-STING signalling pathway, driving the massive release of pro-inflammatory factors by macrophages and ultimately exacerbating joint tissue damage. Experiments have confirmed that targeted knockdown of cGAS or STING proteins effectively blocks the activation of this pathway, significantly reducing the release of inflammatory factors and alleviating joint lesions (Zhang et al., 2026). Experimental studies in mice have shown that the glycyrrhizic acid (GPA) can significantly improve the clinical symptoms of RA. In the pathological microenvironment of RA, exosomes (LPS-exo) released by pro-inflammatory FLS induce M1-polarised macrophages, activate the glycolytic pathway, and cascade-amplify synovial inflammation. Studies have confirmed that GPA can effectively reverse this exosome-mediated vicious cycle: it not only inhibits cellular glycolysis and blocks M1-type polarisation, but also significantly downregulates H3K56a histone lactic acid modification. In-depth mechanistic studies indicate that the aforementioned comprehensive protective effects of GPA depend on its fine regulation of ACLY and HDAC6 expression (Pan et al., 2026). Another study established a RA model in Sprague-Dawley rats to evaluate the pharmacological effects of G-GEB. The research found that the extract of Gastrodia elata Bl., Parishin E (PE), could inhibit abnormal polarization of macrophages by regulating the glycolytic pathway and specifically downregulate the lactylation modification at histone H3 sites (especially H3K18La and H3K27La), thereby effectively halting the pathological progression of RA (Liu X. et al., 2025).

In summary, the metabolite lactate and the lactic acid-mediated modifications it induces represent the core molecular events driving the immunometabolic dysregulation in RA. Consequently, precisely targeting the lactate metabolic network or inhibiting specific lactic acid-mediated modifications has emerged as a key avenue for the future development of innovative therapeutic strategies for RA.

## 5 Clinical implications: targeting the epimetabolic axis

### 5.1 Targeting acetylation modifications

In recent decades, the treatment of RA through the modulation of epigenetic modifications of histones or DNA has gradually attracted widespread attention within the academic community and has become a central research focus in this field. As a reversible epigenetic modification, histone acetylation is a key mechanism regulating chromatin conformation, exerting extensive and precise control over critical biological processes such as gene transcription, the cell cycle and DNA damage repair (Wen et al., 2026). As a central regulatory mechanism for the transcription of inflammatory genes, histone acetylation has become a research hotspot in recent years (Narita et al., 2019). HATs and HDACs function in an antagonistic manner, jointly maintaining a dynamic equilibrium of histone acetylation. In particular, HATs promote chromatin loosening by catalysing acetylation, thereby activating gene transcription. This epigenetic co-regulation not only determines the transcriptional switching, nuclear-cytoplasmic localisation and protein interactions of downstream genes, but also finely regulates cell growth, differentiation and migration, serving as a core mechanism for maintaining cellular homeostasis and organismal health (Sun and Lv, 2026).

Studies have shown that there are significant abnormalities in histone deacetylase (HDAC) expression in FLS from patients with RA. As key epigenetic regulatory factors, HDACs alter chromatin conformation by reducing histone acetylation levels, thereby driving the expression of pathogenic genes in RA (Sun and Lv, 2026). Consequently, HDAC inhibitors (HDACis), currently attracting significant attention as epigenetic modulators, can significantly increase the acetylation levels of histones and non-histone lysine residues by specifically blocking the deacetylation process (Sun et al., 2022). Mechanistically, HDACis inhibit the malignant proliferation and invasion of FLS by modulating chromatin-binding proteins, inducing apoptosis and cell cycle arrest, thereby alleviating the pathological progression of RA (Sun and Lv, 2026). In vivo RA models, they also demonstrate exceptional anti-inflammatory and bone-protective potential, effectively alleviating joint inflammation and curbing the process of bone destruction (Vijaykrishnaraj et al., 2023).

A growing body of research indicates that bioactive compounds derived from various traditional Chinese medicine formulations and their metabolites have been identified as potential modulators of HATs or HDACs (Wen et al., 2026). These active metabolites from traditional Chinese medicine can effectively mediate downstream anti-inflammatory and immunomodulatory effects by specifically regulating the expression or enzymatic activity of HDACs, thereby providing new epigenetic intervention strategies for disease treatment (Wei et al., 2022). In the CIA model rat experiment, the mechanism study revealed that Gardenia glycosides (GE) inhibited the activity of SphK1, thereby down-regulating the S1P/PI3K-Akt signaling axis. This, in turn, suppressed the activation of HDAC3/6 and promoted the acetylation-dependent degradation of HIF-1α. This pathway blockage effectively inhibited VEGF-induced synovial vascularization mediated by S1P and significantly improved the dysfunction of vascular endothelial cells (VECs). This discovery not only confirmed that inhibiting synovial vascularization is an effective approach for treating RA, but also suggested that SphK1 may potentially become a new biomarker for clinical diagnosis and targeted treatment of RA (Li et al., 2026). Other studies have confirmed that resveratrol (Res) can significantly improve arthritis symptoms in CIA and Cit-CIA model mice. At the molecular level, Res inhibits NLRP3 acetylation by activating SIRT1-mediated deacetylation, thereby reducing the secretion of caspase-1 p20 and IL-1β p17, thus blocking LPS-induced NLRP3 inflammasome activation; on the other hand, Res competitively binds to integrin α5β1 (ITGB α5β1) in the presence of ACPA, downregulating Pannexin channel activity and inhibiting ATP release, thereby effectively antagonising ACPA-induced inflammasome activation (Cai et al., 2025). As a classic formula for the clinical relief of RA symptoms, the pharmacological mechanism of Wutou Decoction (WTD) has been shown to be closely related to SIRT1-mediated epigenetic regulation. Studies have shown that WTD exerts significant anti-inflammatory effects by activating the SIRT1 molecule, which in turn downregulates HMGB1 expression and inhibits NF-κB acetylation levels. This finding provides robust molecular biological evidence for the clinical efficacy of traditional Chinese medicine in the treatment of RA (Wen J. et al., 2025). Furthermore, in the experimental study of CIA in mice, studies have confirmed that butyrate significantly inhibits the activity of HDACs in intestinal epithelial cells, whilst overexpression of HDAC3 reverses the upregulation of vitamin D receptor (VDR) and corticosin (CST) expression induced by butyrate. Chromatin immunoprecipitation (ChIP) analysis further demonstrated that butyrate specifically enhances histone acetylation levels in the P3 and P4 regions of the VDR promoter. In summary, intestinal butyrate primarily induces the expression of CST in ileal epithelial cells via the HDAC3-VDR axis, thereby exerting its distal anti-RA effects (Liao et al., 2025).

In summary, acetylation modifications of both histones and non-histones play a key regulatory role in the progression of RA. In the future, elucidating the underlying molecular mechanisms and developing intervention strategies targeting these acetylation modifications, drawing on traditional Chinese medicine, will represent a highly promising area of research in this field.

### 5.2 Targeting methylation modifications

As a key mechanism regulating gene expression, low methylation in the promoter region typically mediates transcriptional activation, whereas high methylation induces gene silencing. This epigenetic process is catalysed by DNA methyltransferases (DNMTs), which transfer a methyl group from SAM to the fifth carbon atom of the cytosine ring. In terms of functional classification, the DNMT family primarily comprises DNMT1, which is responsible for maintaining methylation patterns, and DNMT3a and DNMT3b, which are responsible for *de novo* methylation synthesis; furthermore, the catalytically inactive cofactor DNMT3L acts as a key regulator, significantly enhancing the enzymatic activity of the latter two (Huang et al., 2025).

Epigenetic studies have confirmed that RASFs exhibit significant genome-wide hypomethylation (Mołoń et al., 2026). Throughout the course of RA, high methylation and downregulation of anti-inflammatory genes are frequently observed, thereby maintaining and exacerbating local inflammation (Yang et al., 2022). In contrast, methylation levels in peripheral immune cells still exhibit significant heterogeneity across different studies (Mołoń et al., 2026). By comparing the methylation profiles of synovial cells from RA and osteoarthritis (OA) patients, the study identified a total of 1,859 disease-specific differentially methylated sites (DMPs), of which 60.6% were hypermethylated and 39.4% were hypomethylated (Whitaker et al., 2013). It is worth noting that abnormal DNA methylation profiles are gradually emerging as non-invasive biomarkers for the early diagnosis and prognostic assessment of RA. For example, clinical cohort studies have shown that the methylation level of the DUSP22 gene promoter region in cell-free DNA (cfDNA) from RA patients is significantly lower than that in healthy controls (36.7% ± 3.3% vs. 46.9% ± 2.6%, p = 0.049), and the methylation level of this gene shows a significant downward trend with increasing patient age (Delgado-Cruzata et al., 2026).

It is worth noting that RASFs exhibit characteristic metabolic and epigenetic abnormalities: their intracellular levels of S-adenosylmethionine (SAM), 5-methylcytosine (5 mC) and DNMT1 are simultaneously downregulated, whilst polyamine metabolic pathways are significantly enhanced. This line of evidence suggests that hyperactive polyamine metabolism may drive global hypomethylation of the RASF genome by competitively depleting SAM pools, in conjunction with insufficient DNMT1 expression (Huang et al., 2025). Consequently, reversing and targeting this abnormal DNA methylation state has become a key strategy for intervening in RA. *In vivo* experiments have confirmed that sinapine (SIN) significantly improves paw swelling, reduces the arthritis index, and alleviates synovial inflammation in CIA rats. The molecular mechanism primarily lies in SIN’s ability to induce high DNA methylation at specific sites on the IL-6 promoter, thereby specifically inhibiting the transcription of this pro-inflammatory factor at the epigenetic level, and consequently effectively alleviating the pathological progression of RA (Luo et al., 2026).

A growing body of evidence suggests that m6A RNA methylation plays a pivotal role in regulating immune dysregulation and synovial malignant proliferation in RA. Various natural bioactive compounds and traditional Chinese medicine formulations can exert anti-RA effects by remodelling the m6A methylation profile. Sun et al. conducted a retrospective clinical study involving 2,367 R A patients. The study demonstrated that the Xin Feng Capsules (XFC) significantly increased the m6A methylation level of LINC00968 by inhibiting the catalytic activity of the demethylase ALKBH5, thereby effectively improving clinical symptoms in RA patients. This mechanism further suppresses neutrophil dysfunction and the formation of neutrophil extracellular traps (NETs), breaking the vicious cycle of oxidative stress and inflammatory responses (Sun et al., 2025). Research has confirmed that ginsenoside Rh2-pretreated exosomes (Rh2-pre Exo) can modulate the m6A levels of CCRL2, thereby interfering with the interaction between CCRL2 and TLR4 and blocking the downstream TLR4/MyD88/NF-κB pro-inflammatory signalling pathway, thereby significantly alleviating joint damage in CIA rats (Zhou et al., 2025). Cui et al. established an MH7A cell model by inducing TNF-α and intervened with Astragalosides (AST). The study found that astragaloside A (AST) effectively downregulates the m6A modification levels of WTAP, a component of the m6A methyltransferase complex, and its target gene TRAIL-DR4, whilst simultaneously inhibiting the protein expression of BCL2, BAX and TRAIL-DR4. This combined effect significantly attenuated the proliferative activity of MH7A cells and inhibited pathological synovial hyperplasia by initiating the apoptotic programme (Cui et al., 2025). Furthermore, in the experimental study of CIA in mice, the natural product artemisinin (ATT), by interfering with METTL3-mediated m6A methylation, specifically downregulates the methylation level of ICAM2 mRNA, thereby blocking the migration and invasion of RA-FLS at the post-transcriptional level, providing a novel molecular target for the precision treatment of RA (Wen J. et al., 2025).

In summary, abnormal regulation of DNA and RNA methylation levels plays a pivotal role in the pathological progression of RA. Consequently, elucidating the underlying molecular mechanisms and developing intervention strategies targeting epigenetic methylation based on traditional Chinese medicine represent highly promising avenues of research in this field.

### 5.3 Targeting histone lactylation modifications

Lactic acid was once regarded as the end product of glycolysis, but its bidirectional regulatory role in the pathological microenvironment of arthritis is now attracting significant attention. Under physiological conditions, lactate participates in energy metabolism as a key intermediate in gluconeogenesis and exerts epigenetic regulation via histone lactylation; however, in the pathological processes of OA and RA, locally accumulated lactate transforms from a ‘metabolic marker’ into an ‘inflammatory driver’, significantly exacerbating the inflammatory cascade of arthritis by inducing metabolic reprogramming in synovial and immune cells (Chen et al., 2026). Naughton et al. used magnetic resonance spectroscopy (MRS) to confirm the hypoxic nature of the synovial inflammatory region (Chen et al., 2026). In the RA microenvironment, RASFs exhibit a ‘Warburg effect’ similar to that of tumour cells, whereby abnormally active aerobic glycolysis occurs preferentially even in the presence of sufficient oxygen. This metabolic reprogramming not only drives the malignant proliferation of synovial tissue but also leads to the massive production and accumulation of intracellular lactate (Wang et al., 2022). Monocarboxylate transporters (MCTs) are key molecules governing the transmembrane transport of lactate. Among these, MCT1 (SLC16A1), which possesses high substrate affinity, mediates bidirectional lactate transport across the membrane in a concentration-dependent manner, thereby precisely maintaining lactate homeostasis within and outside the cell (Li et al., 2022). Ultimately, this excreted lactate directly induces acidification of the synovial microenvironment and further permeates across the membrane into surrounding T cells, macrophages and osteoclasts, precisely regulating the local immune microenvironment of the joint through metabolic coupling (Yi et al., 2022).

Abnormally elevated levels of histone acetylation modifications (such as H3K18La) in RA synovial tissue are key factors driving the progression of joint pathology. Experimental studies have shown that downregulating H3K18La levels significantly alleviates joint symptoms in rats with CIA and suppresses the pathogenic potential of TNF-α-induced FLSs. Conversely, the accumulation of H3K18La exacerbates pathological damage. As a central regulatory molecule, the deacetylase SIRT3 is significantly downregulated in peripheral blood mononuclear cells from RA patients; its absence leads to elevated H3K18La levels, thereby activating FLSs and macrophages; conversely, SIRT3 precisely blocks the progression of RA through its specific deacetylation activity (Zheng et al., 2025). In addition to histones, non-histone lactylation is also deeply involved in the immune dysregulation of RA. In primary synovial macrophages from RA patients and CIA mice, dsDNA and lactate levels show a synchronous increase. Accumulated lactate targets the cGAS protein via lactylation, enhancing its stability and inhibiting its degradation, thereby persistently activating the cGAS-STING signalling pathway. The excessive activation of this pathway is key to promoting joint inflammation. Experiments have demonstrated that knocking down cGAS significantly reduces levels of inflammatory cytokines, whilst knocking down STING effectively reverses tissue damage in CIA mice (Zhang et al., 2026).

In response to the abnormal activation of the ‘glycolysis-lactate production-lactylation’ cascade, a variety of natural products and single compounds from traditional Chinese medicine have demonstrated significant potential for targeted intervention. In the CIA model, luteolin (LUT) exhibited significant anti-inflammatory and analgesic effects. The mechanism lies in its ability to effectively inhibit the activity of lactate dehydrogenase A (LDHA), thereby blocking glycolysis-driven histone lactylation (H3K9La), which leads to the transcriptional silencing of the pro-inflammatory factor NFATC2. Through precise regulation of the LDHA-H3K9La-NFATC2 axis, LUT synergistically inhibits the differentiation and migration of Th17 cells (Jiang et al., 2026). In their study, Tan et al. utilised FLSs derived from RA patients to demonstrate that beta-sitosterol can directly bind to and degrade LDHA, thereby downregulating the lactic acid modification of glucose-6-phosphate isomerase (GPI) and reducing its protein stability, thus significantly inhibiting the proliferation, migration and secretion of pro-inflammatory factors (IL-1β, IL-6 and TNF-α) by FLSs (Tan et al., 2025).

Furthermore, addressing the key mechanism by which exosomes secreted by inflammatory FLSs (LPS-exosomes) induce M1 macrophage polarisation and synovial inflammation, geniposide (GPA) blocked LPS-exo-induced M1 polarisation by inhibiting cellular glycolysis, whilst simultaneously downregulating H3K56La modification, thereby suppressing inflammatory progression through both metabolic and epigenetic mechanisms (Pan et al., 2026). *In vitro* studies demonstrated that 10–20 μM gastrodin (GAS) significantly downregulated the expression of pro-inflammatory factors (IL-6, MMP1, MMP13) in LPS-induced FLSs and THP-1 macrophages by inhibiting glycolysis and lactate production. Mechanistic analysis indicates that GAS reduces histone H3K9La levels by inducing the instability of the acetyltransferase KAT8. *In vivo* experiments further validated that 20 mg/kg of GAS effectively alleviates joint swelling and synovial hyperplasia in rats, and this therapeutic effect is closely associated with reduced levels of H3K9La and IL-6 (Dai et al., 2025). Furthermore, as another key component of Gastrodia elata, Parishin E (PE) blocks hyperglycolysis by downregulating the expression of hexokinase 2 (HK2) and LDHA, thereby inhibiting the pro-inflammatory polarisation of macrophages and specifically suppressing the lactylation modification of specific histone H3 sites (H3K18La and H3K27La), thereby halting the pathological progression of RA at the ‘metabolic-epigenetic’ axis (Liu X. et al., 2025).

In summary, lactic acid accumulation and the epigenetic lactic acid-mediated modifications it induces play a pivotal role in the pathological progression of RA. Therefore, this article systematically analyzed the micro-regulatory network of the core metabolites in RA, and prospectively proposed a traditional Chinese medicine intervention strategy of reshaping the epigenetic map and targeting the inhibition of abnormal epigenetic modifications, providing a highly promising research direction for exploring new clinical treatment paths for RA (Table 2).

**TABLE 2:** Therapeutic strategies and mechanisms for epigenetic modifications in RA.

Epigenetic regulatory mechanisms	Representative intervention measures	Key metabolic/mechanistic targets	Comparison of key experimental parameters	References
Targeteing acetylation modification	Gardenia glycosides (GE)	Inhibit the activity of SphK1,Downregulate the S1P/PI3K-Akt signaling axis	Inhibit synovial vascularization and improve the function of VEC	Li et al. (2026)
	Resveratrol (Res)	Activate SIRT1,Inhibit NLRP3 acetylation Competitively bind to ITGB α5β1,Reduce Pannexin	Reduce the secretion of caspase-1 p20 and IL-1β p17,Inhibit inflammasome activation	Cai et al. (2025)
	Wutou Decoction (WTD)	Activate SIRT1,Downregulate HMGB1 and inhibit the acetylation of NF-κB	Anti-inflammatory	Wen J. et al. (2025)
	Butyrate	Inhibite the activity of HDAC, Increase VDR and CST	Anti-inflammatory	Liao et al. (2025)
Targeteing Methylation Modification	Sinapine (SIN)	Induce IL-6 promoter DNA hypermethylation	Improve paw swelling, reduce arthritis index and alleviate synovial inflammation	Luo et al. (2026)
	Xin Feng Capsules (XFC)	Inhibit the catalytic activity of ALKBH5,Increase the m6A methylation level of LINC00968	Block the vicious cycle of oxidative stress and inflammatory response	Sun et al. (2025)
	Rh2-pre Exo	Regulate m6A levels of CCRL2,Block the TLR4/MyD88/NF-κB signaling cascade	Relieve joint injuries	Zhou et al. (2025)
	Astragaloside A (AST)	Reduce the m6A modification levels of WTAP and its target gene TRAIL-DR4,Inhibit the protein expression of BCL2, BAX and TRAIL-DR4	attenuate the proliferative activity of MH7A cells and inhibit pathological synovial hyperplasia	Cui et al. (2025)
	Artemisinin (ATT)	Intervene in METTL3-mediated m6A methylation modification, Reduce the methylation level of ICAM2 mRNA	Block the migration and invasion of RA-FLS	Wen J. et al. (2025)
Targeting histone lactylation modification	Luteolin (LUT)	Inhibite the LDHA-H3K9La-NFATC2 axis, Inhibite the differentiation and migration of Th17 cells	Anti-inflammatory and analgesic properties	Jiang et al. (2026)
	Beta sitosterol	Degradate LDHA, Reduce the lactylation modification level of GPI and decrease its protein stability	Inhibit the proliferation, migration of FLSs and the secretion of pro-inflammatory factors (IL-1β, IL-6, and TNF-α)	Tan et al. (2025)
	Geniposide (GPA)	Inhibit cellular glycolysis, block M1-type polarization, and downregulate H3K56La modification	Inhibit inflammation	Pan et al. (2026)
	Gastrodin (GAS)	Inhibit glycolysis and lactate production, Reduce the level of histone H3K9La	Reduce the expression of pro-inflammatory factors (IL-6, MMP1, MMP13)	Dai et al. (2025)
​	Parishin E (PE)	Reduce the expression of HK2 and LDHA, Inhibit the polarization of M1 macrophages and suppress the lactylation modifications of H3K18La and H3K27La	Inhibit inflammation	Liu X. et al. (2025)

Although the ‘epigenetic metabolic axis’ demonstrates vast therapeutic potential, the transition from preclinical research to clinical application still faces numerous ‘valleys of death’, and the translational process urgently requires a systematic review of the following barriers: (1) Target specificity and systemic risks: Existing pan-HDAC inhibitors (such as SAHA) or DNMT inhibitors (such as azacitidine), due to their lack of cellular selectivity, are highly prone to disrupting the epigenetic homeostasis of healthy tissues throughout the body whilst inhibiting the expression of inflammatory genes in RA FLS, leading to serious side effects such as myelosuppression, gastrointestinal damage and secondary tumours. Therefore, the development of precision delivery systems based on synovial-targeting nanoparticles or FLS-specific ligands—which confine modulators to the microenvironment of the affected joint—is the only viable path to reducing off-target toxicity. (2) Variations in response due to disease heterogeneity: The molecular pathology of RA is highly complex, and not all patients exhibit the classic ‘Huabao effect’. Intervention strategies based on the ‘glycolysis–lactation’ axis are effective only for the metabolically active subtype of RA and have limited efficacy in patients where other pathogenic pathways predominate. Clinical translation should incorporate a ‘companion diagnostics’ model, utilising biomarkers such as serum lactate and specific histone modifications to precisely stratify patients and enable personalised treatment. (3) Challenges regarding pharmacological homeostasis and long-term efficacy: Metabolic-epigenetic regulation acts directly at the source of gene transcription; whilst it holds potential for disease modification, it is susceptible to limitations imposed by ‘therapeutic resilience’. If pathogenic environmental stresses (such as hypoxia or microbiome dysbiosis) persist, the ‘metabolic memory’ of pathogenic cells may lead to a rapid rebound of the pathological phenotype following discontinuation of treatment. Developing a combination therapy comprising ‘metabolic modulators plus conventional immunotherapeutics’ is not only intended to reset the cellular pathological phenotype but is also a key strategic element in ensuring long-term efficacy. (4) Physiological safety under long-term intervention: Given the fundamental role of epigenetic regulation in physiological functions, long-term intervention may inadvertently compromise the immunostative functions of regulatory T cells (Tregs) or reparative M2 macrophages. There is an urgent need to conduct prospective safety evaluations using organoid-on-a-chip or synovial tissue organ culture models to thoroughly assess the impact of metabolic interventions on the intrinsic regenerative potential of joint tissues and the risks of unintended immunosuppression.

## 6 Limitations and future perspectives

Current Limitations in Understanding the Epimetabolic Axis: Although recent research has begun to outline the role of the ‘metabolic-epigenetic’ axis in the pathogenesis of RA, significant gaps in our understanding remain: (1) The dilemma of establishing causality: Much of the current literature relies on descriptions of correlations observed in pathological states. It remains unclear whether these metabolic reprogramming events are the ‘primary drivers’ of inflammatory phenotypic changes, or merely ‘metabolic signatures’ resulting from alterations in the inflammatory microenvironment. This lack of causal clarity hinders our ability to identify the most effective intervention points. (2) Lack of spatial resolution: RA synovial tissue is a complex spatial system comprising various immune and stromal cells. Existing omics studies predominantly use ‘bulk tissue’ analysis, obscuring the subtle differences in metabolic flux between different cell subsets and their spatially specific epigenetic modification patterns. (3) ‘Non-specific’ accumulation of metabolites: Current observations of intermediate metabolites (such as lactate and succinate) are largely based on extracellular environments or overall intracellular concentrations. However, the true pathological significance lies in metabolite concentrations within the nuclear microenvironment; we currently lack the technical means to accurately detect nuclear metabolic fluxes and the specific histone modifications they drive.

Future Research Directions and Emerging Technologies: To address the challenges outlined above, future research must urgently shift towards precision and multidimensional approaches: (1) Single-cell spatial metabolic-epigenomic multi-omics: Future research should combine spatial transcriptomics with high-resolution mass spectrometry imaging to elucidate the correlation between metabolite distribution and chromatin modification states within different microtissues of the synovial microenvironment in RA, thereby enabling the precise identification of pathogenic mechanisms. (2) Real-time Metabolic Monitoring and Imaging (Live-cell Metabolite Imaging): Fluorescent probes specific to metabolites such as lactate and acetyl-CoA, as well as their corresponding modifying enzymes, should be developed to dynamically observe the transient interactions between intracellular metabolite concentrations and histone modifications in RA-FLSs and immune cells under inflammatory stimulation. (3) Identification and Targeting of ‘Reader’ Proteins: How are novel modifications, such as lactylation, recognised by specific proteins and translated into instructions for gene transcription? Future research will require large-scale screening for ‘readers’ of lactylation modifications, which will be key to discovering entirely new drug targets. In summary, the pathological essence of RA has evolved from a simple immune dysregulation to a self-sustaining system characterised by ‘metabolic-epigenetic’ coupling. However, current translational approaches face significant limitations: existing interventions (such as HDAC inhibitors) often exhibit multi-target toxicity. Future research must shift its focus to ‘cell-specific delivery systems’; otherwise, epigenetic interventions will remain difficult to translate from the laboratory to the clinical setting. We need to move from ‘identifying relevant markers’ to ‘validating functional interventions’.
